# Humidity-sensitive chemoelectric flexible sensors based on metal-air redox reaction for health management

**DOI:** 10.1038/s41467-022-33133-y

**Published:** 2022-09-15

**Authors:** Shuo Li, Yong Zhang, Xiaoping Liang, Haomin Wang, Haojie Lu, Mengjia Zhu, Huimin Wang, Mingchao Zhang, Xinping Qiu, Yafeng Song, Yingying Zhang

**Affiliations:** 1 Key Laboratory of Organic Optoelectronics and Molecular Engineering of the Ministry of Education, Department of Chemistry, Tsinghua University, Beijing, 100084 P. R. China; 2 Institute of Sport and Health Science, Beijing Sport University, Beijing, 100084 P. R. China

**Keywords:** Nanoscale materials, Sensors, Sensors and biosensors

## Abstract

Numerous studies have shown flexible electronics play important roles in health management. The way of power supply is always an essential factor of devices and self-powered ones are very attractive because of the fabrication easiness, usage comfort and aesthetics of the system. In this work, based on the metal-air redox reaction, which is usually used in designing metal-air batteries, we design a self-powered chemoelectric humidity sensor where a silk fibroin (SF) and LiBr gel matrix containing parallel aligned graphene oxide (GO) flakes serve as the electrolyte. The abundant hydrophilic groups in GO/SF and the hygroscopicity of LiBr lead to tight dependence of the output current on the humidity, enabling the sensor high sensitivity (0.09 μA/s/1%), fast response (1.05 s) and quick recovery (0.80 s). As proofs of concept, we design an all-in-one respiratory monitoring-diagnosing-treatment system and a non-contact human-machine interface, demonstrating the applications of the chemoelectric humidity sensor in health management.

## Abstract (web summary)

Self-powered sensors are of interest in wearable technology and other applications. Here, the authors report on the creation of a metal-air redox reaction humidity sensor where the conductance and charge generated is influenced by the amount of absorbed water and demonstrate application in respiration monitoring.

## Introduction

Numerous studies have shown flexible electronics can be applied in health management, and hold great potential to revolutionize people’s lifestyles^1–7^. For example, monitoring respiration, including the frequency, rhythm, and intensity of respiration, is very important for diagnosing respiratory diseases and preventing respiratory failure^8–14^. Currently, most of the reported flexible electronics are equipped with separate power supply devices, which need to be assembled through external circuits^15–18^. The complicated circuit design and wire generate high requirements for device structure and integration techniques, which inevitably affect the comfort and esthetics, and increase the probability of system failure. Therefore, it is highly desirable to develop flexible electronics with integrated energy supply functions^19,20^. In the last decade, self-powered electronics, including self-powered sensors, pacemakers, etc., have drawn great interest and been going forward obviously^21–23^. Such electronics can continuously monitor physiological signals or provide therapy without the need for external power devices and bulky wires.

Pioneer researchers have reported self-powered sensors based on various mechanisms, including triboelectric, piezoelectric, thermoelectric, and hydroelectric, which can be driven by friction, compression, temperature gradient, and humidity gradient and thus do not require other power supply^24–30^. Being different from the above phenomenon, the metal-air redox reaction, which is usually used in high-performance metal-air batteries, can convert chemical energy into electrical energy and does not require external stimulation, showing advantages in achieving high stability^31–34^. Previous works have demonstrated that the output performance of a metal-air battery can be affected by external stimuli (e.g., pressure, light, glucose, NO_2_)^35–38^, showing the potential that a self-powered humidity sensor may be realized if humidity can have an obvious impact on the redox reaction in a metal-air battery. Compared to other reported self-powered sensors, the sensor based on metal-air batteries derives its energy from the chemical energy of the anode. Thus, this kind of self-powered sensor may generate a stable signal response without any external energy input.

In this work, we developed a high-performance self-powered chemoelectric humidity (CEH) sensor based on the metal-air redox reaction and demonstrated its potential application in remote health management and medical treatment. The sensor is composed of graphene oxide (GO)/silk fibroin (SF)/LiBr electrolyte gel layer sandwiched between graphite paper and aluminum foil. Since both GO and SF have abundant hydrophilic groups and LiBr has good hygroscopicity, the redox reaction in this structure is highly sensitive to humidity. The humidity can be monitored by measuring the short-circuit current of the sensor without the need for an additional power supply, which depends on the ion mobility of the electrolyte. The GO flakes are parallelly aligned in SF gels, hindering fast ion mobility and thus leading to a long lifetime of the sensor. The sensor showed good performances including quick response (1.05 s), fast recovery (0.80 s), and high sensitivity (0.09 μA/s/1%) in a wide relative humidity (RH) range (11–84%). Based on the excellent performance of the sensor, we designed an integrated respiratory monitoring-diagnosing-treatment system and non-contact human–machine interfaces, demonstrating its promising application in health management, including infection prevention, disease diagnosis, and medical treatment.

## Results

### Preparation of the self-powered CEH sensor

**Fig. 1:**
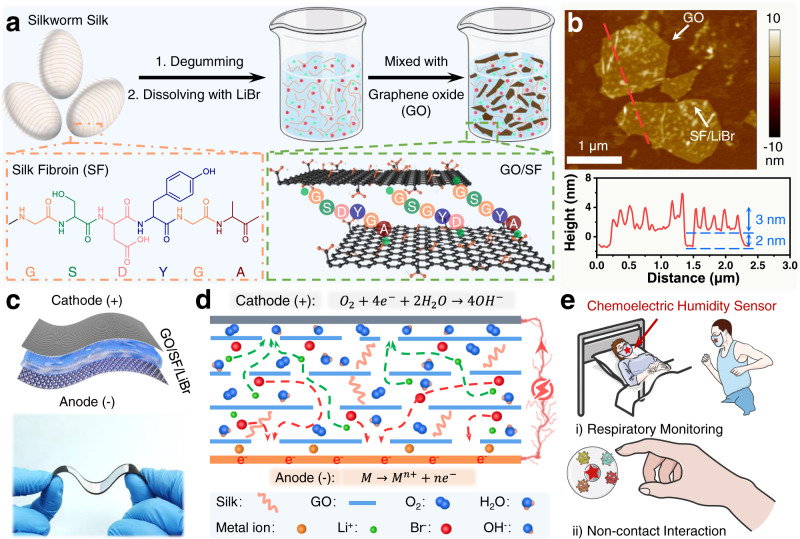
The preparation process and potential applications of graphene oxide (GO)/silk fibroin (SF)/LiBr ink and the obtained chemoelectric humidity (CEH) sensors. **a** Schematic illustration showing the preparation process of GO/SF/LiBr ink and the interaction between SF and GO. **b** Atomic force microscope image showing the morphology and profile of GO/SF/LiBr flakes. The experiment was repeated three times independently with similar results. **c** Schematic illustration and photo image of the humidity sensors. **d** Working mechanism of the chemoelectric sensor based on metal-air redox reaction. **e** Potential applications of the sensors in health management.

The electrolyte layer of the sensor was prepared from a rational designed GO/SF/LiBr ink. Figure 1a illustrates the preparation process of the ink, which is composed of GO and natural silkworm SF dissolved in LiBr aqueous solution. SF is a natural biomacromolecule originating from silk cocoons and is composed of 18 kinds of amino acids. Among them, 22% of the amino acids are polar amino acids (e.g., sericin and tyrosine), which are rich in oxygen-containing groups^39^. The X-ray photoelectron spectrum (XPS) of GO shows that the contents of C and O are about 68.5 and 31.5%, respectively, and the GO is rich in oxygen-containing functional groups (e.g., hydroxyl, carboxyl, and carbonyl) (Supplementary Fig. 1). Therefore, SF can form strong hydrogen bonds and π–π interactions with GO^40,41^. The synergy of hydrogen bonds and π–π interactions endows the GO/SF/LiBr ink with good uniformity, certain structural stability, and good processability (Supplementary Fig. 2). We characterized the structure of the GO/SF/LiBr flakes using transmission electron microscopy (TEM) and atomic force microscopy (AFM). GO presented a sheet-like structure, and the SF/LiBr were adsorbed on the GO sheet (Supplementary Fig. 3). The GO has a thickness of around 2 nm and the height of SF/LiBr adsorbed on the GO is about 3 nm (Fig. 1b and Supplementary Fig. 4). In addition, we compared the intensity ratio of D band to G band (*I*_D_/*I*_G_) of the Raman spectra of GO and GO/SF/LiBr composites and found it increased from 0.97 to 1.05 (Supplementary Fig. 5), indicating the absorption of SF/LiBr on GO^42^. Therefore, GO can be uniformly dispersed in the SF solution at a concentration of up to 10 wt%. Notably, the viscosity of GO/SF/LiBr ink increases as the content of GO increases (Supplementary Fig. 6), enabling the coating of the ink on various substrates through various techniques, e.g., direct writing, screen printing, and extrusion printing (Supplementary Fig. 7).

Figure 1c and Supplementary Fig 8 shows the illustrated structure and the image of the flexible chemoelectric sensor based on the metal-air redox reaction. The GO/SF/LiBr electrolyte layer is sandwiched between graphite paper (cathode layer) and aluminum foil (anode layer). A GO/SF/LiBr ink containing 7.5 wt% GO was used for the fabrication of the sensor unless otherwise noted. The cryo-scanning electron microscopy (Cryo-SEM) image (Supplementary Fig. 9) of a GO/SF/LiBr film shows that there are many pores, which enable the rapid exchange of water molecules and oxygen. Besides, the GO sheets form layered structures, which can be ascribed to the presence of capillary force, hindering fast ion migration. In addition, the abundant oxygen-containing functional groups and hydrophilic groups in GO and SF endow the electrolyte layer with excellent hydrophilicity. LiBr also has excellent hygroscopicity. At the same time, LiBr provides mobile ions. The synergy of the above factors leads to an excellent humidity response of the CEH sensor. In a completely dry environment, the open-circuit voltage of the CEH sensor was measured as about 0 V, which provided the possibility for its long-term storage. As a comparison, in a humid environment (30% RH), the open-circuit voltage of the sensor was about 0.9 V. (Supplementary Fig. 10).

Figure 1d illustrates the working mechanism of the chemoelectric sensors. When the sensor is in a humid environment, the GO/SF/LiBr layer absorbs the water molecules in the air and the LiBr can dissociate into Li^+^ and Br^−^. The metal anode can lose electrons and convert to metal ions. Graphite paper has certain oxygen reduction reaction activity (Supplementary Fig. 11), which can be attributed to the doping of heteroatoms (Supplementary Fig. 12)^43,44^. Therefore, the oxygen will get electrons, which transfer through the external circuit, on the surface of the graphite paper. The above reactions can be expressed as the following:

1 $${{\mbox{M}}}\to {{{\mbox{M}}}}^{{{\mbox{n+}}}}{{\mbox{+n}}}{{{\mbox{e}}}}^{{{\mbox{-}}}}$$

2 $${{{\mbox{O}}}}_{2}{{\mbox{+}}}4{{{\mbox{e}}}}^{{{\mbox{-}}}}{{\mbox{+}}}2{{{\mbox{H}}}}_{2}{{\mbox{O}}}\to 4{{{\mbox{OH}}}}^{{{\mbox{-}}}}$$

where M stands for the active metal in the anode. In the GO/SF/LiBr layer, the Li^+^ and Br^−^ transfer to the cathode and anode, respectively. The ion mobility in the GO/SF/LiBr electrolyte will be affected by the quantity of the absorbed water. Therefore, the measured value of the short-circuit current depends on the humidity, enabling it to work as a self-powered humidity sensor. The obtained CEH sensor shows excellent performance and can be used for non-contact detection of humidity variation, enabling applications in health monitoring (such as respiratory disease diagnosis) and non-contact human–machine interaction (such as non-contact controllers for light switches and elevators) (Fig. 1e).

### Characterization of the self-powered CEH sensor

**Fig. 2:**
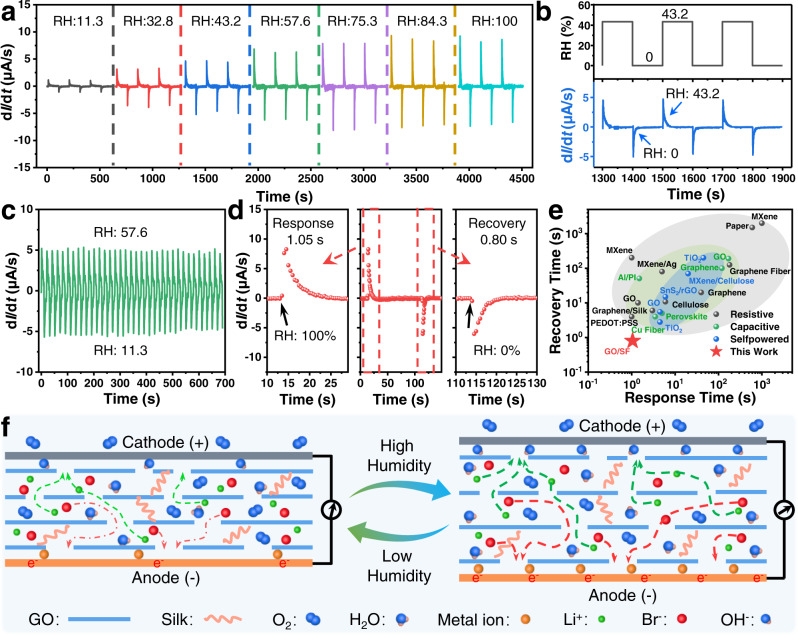
Performance of the self-powered chemoelectric humidity (CEH) sensors. **a** The current change rate (CCR) of the CEH sensor under different relative humidity (RH). **b** The CCR (blue) of the sensor to switch RH (black) between 0 and 43.2%. **c** The CCR of the humidity sensor to cyclic RH changes between 11.3–57.6%, showing high reliability. **d** The response and recovery time to 0–100% RH switch. **e** Comparison of the response time and recovery time of our CEH sensor with previously reported humidity sensors, showing an excellent response, and recovery performance of our sensor (Resistive: MXene^61^, Paper^9^, MXene^62^, MXene/Ag^63^, Graphene^10^, Graphene Fiber^64^, GO^46^, Graphene/Silk^12^, PEDOT:PSS^65^, Cellulose;^49^ Capacitive: GO^66^, Graphene^67^, Al/PI^6^, Perovskite^68^, Cu Fiber;^69^ Self-powered: TiO_2_^29^, MXene/Cellulose^70^, SnS_2_/rGO^28^, GO^71^, and TiO_2_^72^). GO graphene oxide, PEDOT:PSS poly (3,4-ethylenedioxythiophene):poly (styrenesulfonate), PI polyimide, rGO reduced graphene oxide. **f** Schematic illustration showing the response of the structure of the CEH sensors to humidity based on metal-air redox reaction.

We characterized the performance of the CEH sensor by finely tuning the humidity (Supplementary Fig. 13). The environment of the CEH sensor was switched between dry air and humid air with different RH. The testing temperature is 25 °C unless otherwise noted. Figure 2a,  b show the current change rate (CCR, d*I*/d*t*) of the sensor under different RH. The CCR increases from 1.16 to 8.44 μA/s when the RH varies from 11.3% to 84.3%, indicating that the CEH sensor can detect variable humidity with high sensitivity (0.0933 μA/s/1%) in a wide range (Supplementary Fig. 14). Besides, the change of the current of the CEH sensor under different RH was also collected (Supplementary Fig. 15), indicating that the sensor can also be used to detect stable humidity in a wide range.

The CEH sensor has good reliability and fast response. As shown in Fig. 2c, its response is highly reliable when the RH was switched between 57.6% and 11.3%. Besides, the response and recovery time to 0–100% RH switch are 1.05 and 0.80 s (Fig. 2d), respectively. We compared the response time and recovery time of our CEH sensor with the reported flexible resistive, capacitive, and self-powered humidity sensors, as shown in Fig. 2e. The CEH sensor has a short response time and recovery time, laying a foundation for applications in the fields of respiratory monitoring and human–machine interaction (Supplementary Table 1). These results prove the excellent performance of the CEH sensors, including fast response time (1.05 s) and recovery time (0.80 s), and high sensitivity (0.0933 μA/s/1%) in a wide RH working range (11.3–84.3%).

We further explored the effects of temperature, pressure, and anode materials on the humidity sensor. When the temperature increases from 0 to 80 °C, the open-circuit voltage of the sensor increases from 1.12 to 1.29 V (Supplementary Fig. 16). The sensor exhibits significant and stable humidity response signals in the temperature range of 0 to 80 °C, indicating that it can serve as a reliable sensor in a wide temperature range. Besides, when the air pressure is changed from 1 to 0.01 atm, the fluctuation of open-circuit voltage is less than 1% (Supplementary Fig. 17), which is consistent with theoretical analysis (Supplementary Note 1), indicating that air pressure and O_2_ concentration has an ignorable influence on the performance of the CEH sensor. Therefore, slight changes in O_2_ concentration during respiration do not affect the performance of respiration monitoring. In addition, we also measured the open-circuit voltage and sensing performance of CEH sensors using Cu foil, Cu wire, and Cu mesh as the anodes (Supplementary Fig. 18). The results showed that the sensors with Cu anode, despite the morphology, also have stable humidity responses, indicating that CEH sensors can employ other types of metal anodes.

Furthermore, we investigated the stability of the CEH sensor over time and mechanical stability. The average consumption of the anode metal (Al) for 12 h is about 0.18 mg/cm^2^, which costs only $ 0.01/m^2^/d with reference to the commercial price list in 1688.com ($ 2.82/kg), showing a low cost (Supplementary Fig. 19)^45^. The open-circuit voltage of the sensor showed ignorable decrement within the first 36 h of operation and then gradually decayed from 1.24 to 0.51 V in the second 36 h (Supplementary Fig. 20). Accordingly, the response of the sensor also gradually decreased with time, but can still respond effectively to humidity changes after 72 h of continuous operation, indicating the CEH sensor has a service life of up to 72 h (Supplementary Fig. 21). Once the sensor fails, it can be easily regenerated by replacing the used anode metal and electrolyte material with fresh ones, and the sensing performance of the regenerated sensor can be restored to the original level. Besides, after 180 days of storage in a dry environment, the CEH sensor still shows a stable humidity response (Supplementary Fig. 22), indicating long-term storage stability. In addition, the load-deformation curves of the sensor during 300 bending cycles (Supplementary Fig. 23) were recorded and the sensing performance before and after the bending were compared(Supplementary Fig. 24). The results indicate that the sensor has good flexibility and mechanical stability.

### Working mechanism of the self-powered CEH sensors

We proposed the working mechanism of this CEH sensor, which is illustrated in Fig. 2f. Basically, the working mechanism of the sensor is tightly related to the metal-air redox reaction, which is affected by humidity. Active metal in the anode easily loses electrons and becomes metal ions. Therefore, these electrons will transfer to the cathode (graphite paper) and reduce the oxygen dissolved in the GO/SF/LiBr electrolyte, leading to an electric field. Then, ions in the GO/SF/LiBr electrolyte, such as Li^+^ and Br^−^, will migrate directionally in the electric field, and current will be generated in the external circuit at the same time. The metal-air redox reaction provides an energy basis for self-powered humidity sensors.

The response of the sensor to humidity significantly depends on the adsorption of water molecules in the GO/SF/LiBr electrolyte layer. According to the widely reported adsorption theory, water molecules bind hydrophilic sites (e.g., hydroxyl and carboxyl groups) in SF and GO through hydrogen bonds^46–48^. More water molecules will be absorbed under higher humidity, resulting in the swelling of the GO/SF/LiBr and reduction of the transmission steric hindrance to ions. In addition, absorption of water also increases the hydration and mobility of ions^49,50^. The synergy of these two effects will lead to an increase in the ion mobility in the GO/SF/LiBr electrolyte with increased humidity, corresponding to an internal resistance decrease and external current increase. The CCR first increases from 0 to a positive value, corresponding to the rapid absorption of water molecules on the GO/SF/LiBr electrolyte. Then, the CCR gradually returns to 0, indicating the absorption gradually reaching equilibrium. The CCR value is closely related to the water molecules’ adsorption and desorption speeds, which can be affected by external environmental humidity and temperature. Specifically, high humidity will accelerate the adsorption speed of water molecules, resulting in an increase in CCR. Under a certain humidity, the desorption speed of water molecules increases with temperature. Therefore, when the temperature is too high (above 70 °C), the desorption of water molecules is significantly accelerated, leading to a decrease in the total amount of absorbed water and a reduction in the CCR of the sensor.

Our experimental results are in good consistence with the above proposed mechanism. The water contact angle of the GO/SF/LiBr surface was 18° (Supplementary Fig. 25), indicating the excellent hydrophilicity of the GO/SF/LiBr electrolyte. We measured the water content and relative resistance change (Δ*R*/*R*_*0*_) of a GO/SF/LiBr film under different RH. The water content of a GO/SF film (10 mm × 10 mm × 0.1 mm) increased from 33.76% to 66.54% as measured by an analytical balance and its Δ*R*/*R*_*0*_ decreased from −88.63% to −98.50% when the RH varies from 11.3% to 84.3% (Supplementary Fig. 26). Besides, the water content of GO/SF/LiBr at 11.3% RH was also characterized using thermogravimetric analysis, which is in consistence with the above results (Supplementary Fig. 27). The above results prove that when the sensor is exposed to moist air, the electrolyte tends to quickly absorb water from the air, leading to obvious changes in the resistance.

### Respiratory monitoring-diagnosing-treatment based on self-powered CEH sensors

As an indispensable physiological behavior of humans, breathing maintains human life by ensuring the exchange of oxygen and carbon dioxide. However, respiratory diseases such as sleep apnea syndrome (SAS), asthma, and pneumonia, can severely endanger life within a few minutes. For example, SAS refers to the repeated occurrence of apnea or hypopnea symptoms due to various reasons, leading to brain hypoxia, lethargy, hypertension, coronary heart disease, mental disorders, diabetes, and other diseases^51,52^. Therefore, monitoring respiration through non-invasive sensors is particularly necessary for health management. Due to the excellent sensing performance, especially the fast response and recovery, the CEH sensor shows great potential in health management and telemedicine, including respiratory monitoring, SAS diagnosing, and treatment.

**Fig. 3:**
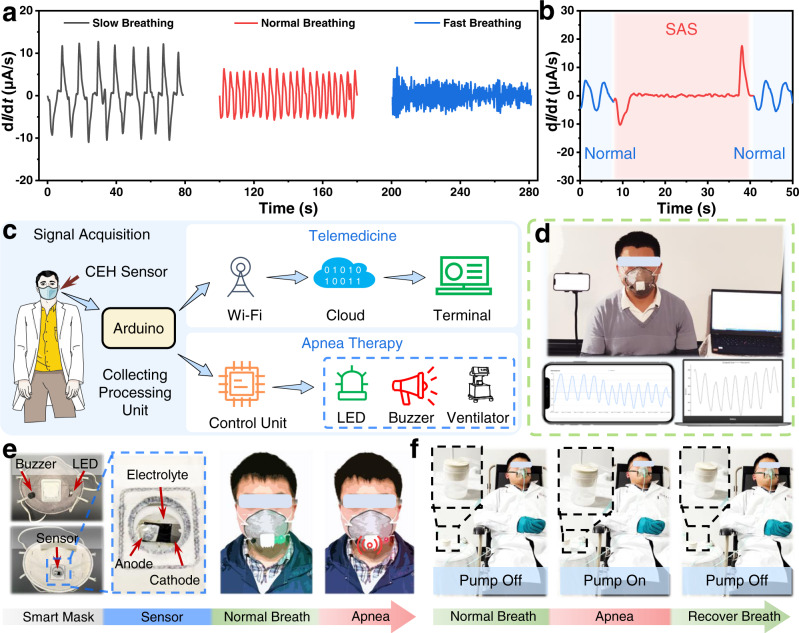
Applications of the self-powered chemoelectric humidity (CEH) sensors in respiratory monitoring, telemedicine, sleep apnea syndrome (SAS) diagnosing, and treatment. **a** Signals of breathing at different frequencies detected by the CEH sensor. **b** A typical breathing signal of patients with SAS (Blue: Normal, Red: SAS). **c** Schematic illustration of the all-in-one respiratory monitoring-diagnosing-treatment system based on the CEH sensors. **d** Photos of uploading a patient breathing data to remote terminals (phone and computer) with wireless networks in real-time. **e** Photos of a smart mask integrated with a CEH sensor, buzzer, and a light-emitting diode (LED), which can alert people with SAS with a buzzer and LED. **f** Photos of a SAS diagnosing-treating system with an integrated CEH sensor that controls the ventilator in time by monitoring the breathing status.

Figure 3a shows the response signals of the CEH sensor at different respiratory frequencies. The sensor can recognize breath with different frequencies, including slow, normal, and fast breathing, corresponding to relaxed, normal, and exercise states, respectively. Even with a fast respiratory rate of up to 60 breaths per minute, the sensor still shows excellent breathing monitoring performance (Supplementary Fig. 28a). In addition, the CEH sensor can be used to distinguish inhalation and exhalation (Supplementary Fig. 28b). Typical breathing signals of patients with SAS were shown in Fig. 3b. The signals of normal breath and apnea are marked in blue and red, respectively. Compared with normal signals, the CCR of the apnea process is almost zero, which can be used for the diagnosis of apnea syndrome.

We designed an all-in-one respiratory monitoring-diagnosing-treatment system and demonstrated its application. The system is composed of a signal collecting unit (humidity sensor), a signal processing unit (Arduino), a signal transmission unit (wireless connection module), a control unit (Arduino), and an output unit (light-emitting diode (LED), buzzer, and air pump) with multi-working modes (Fig. 3c and Supplementary Figs. 29,  30). The system can collect respiratory data and upload it to the network in real-time through wireless networks (Fig. 3d and Supplementary Movie 1), which can be used for telemedicine. In addition, the system can monitor respiratory frequency. The blinking frequency of LED increases with the respiratory frequency (Supplementary Movie 2), which can be used to predict physical state (e.g., relaxation or tension).

Furtherly, we demonstrated the application of the humidity sensors in the diagnosis and treatment of sleep apnea syndrome (Figs. 3e, f). An important way to treat SAS is to intervene in time when the patient has apnea, such as waking the patient to resume active breathing. When the SAS diagnosis and treatment system recognizes that the patient has apnea, the LED will emit red, and the buzzer will alarm, to alert people to avoid further dangers (Fig. 3e, Supplementary Fig 29b, and Supplementary Movie 3).

Another important way to treat SAS is through ventilator-assisted ventilation therapy. Currently, most of the current ventilators can only work continuously with the set parameters, which cannot be adjusted in time by monitoring the breathing status of the patient. In contrast, the designed SAS diagnosis and treatment system can actively treat SAS with a high degree of comfort (Supplementary Fig. 29c). When apnea is detected, the air pump starts to work, and after the patient resumes active breathing, the air pump is automatically turned off (Fig. 3f and Supplementary Movie 4).

### Non-contact human–machine interface based on self-powered CEH sensors

At present, most of the human–machine interaction is accomplished through contact, such as mobile phone touch screens, computer keyboards, mouses, elevator switches, etc. However, those contact human–machine interaction tends to increase risks such as the spread of viruses and bacteria^10,53,54^. In addition, emerging technologies such as augmented reality put forward new requirements for the interactive process^55^. We measured the humidity on the surface of human fingers. The result shows that the humidity on the finger surface decreased with distance (Supplementary Fig. 31), which is consistent with reported works^10^, endowing the possibility of the CEH sensor for human–machine interaction. Therefore, non-contact human–machine interfaces are highly desirable, especially in the context of the COVID-19 epidemic^46,56–58^. Based on the high-sensitive CEH sensor, we designed a non-contact human–machine interface, which can detect the approach of fingers due to the inherent humidity field of human skin, thus, avoiding virus transmission and bacterial infection induced by contact.

**Fig. 4:**
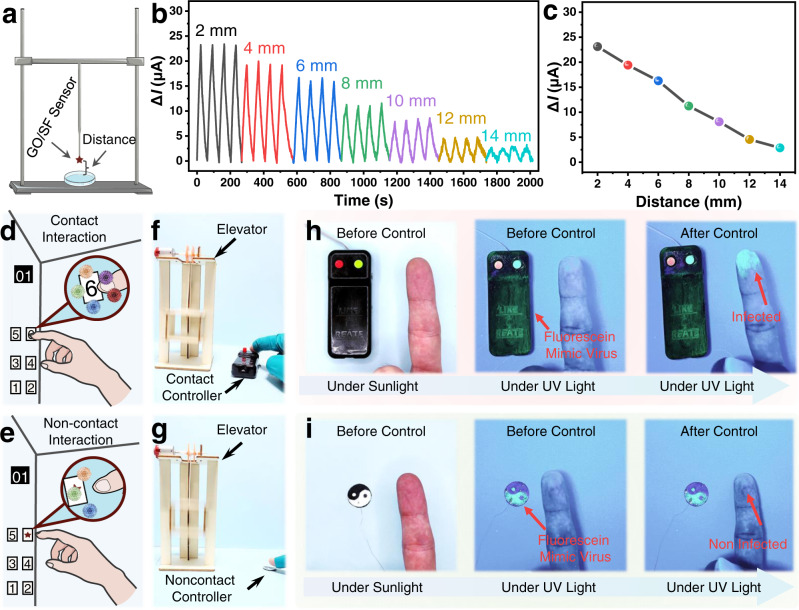
Applications of the self-powered chemoelectric humidity (CEH) sensors in non-contact human–machine interaction. **a** Schematic illustration of a non-contact distance sensing testing set. GO graphene oxide, SF silk fibroin. **b**, **c** The short-circuit current change (Δ*I*) of the CEH sensor at different distances above the water surface (one sensor was measured repeatedly four times and error bars represent standard errors). **d**, **e** Schematic illustration showing conventional contact human–machine interaction with the risk of virus transmission (**d**) and non-contact human–machine interaction without risk of virus transmission (**e**). **f**, **g** Photo of conventional contact controller (**f**) and non-contact human–machine interface (**g**). **h**, **i** Optical photos (under sunlight) and fluorescence photos (under 365 nm ultraviolet (UV) light) of a finger before and after operating the contact button controller (**h**) and non-contact controller (**i**).

As illustrated in Fig. 4a, a humidity sensor was placed at different heights above the water surface to verify its ability to be used as non-contact human–machine interfaces. The water surface can be considered as a humidity source of 100% RH. Figure 4b, c show the short-circuit current change (Δ*I*) with different heights above the water surface, indicating the high stability and reliability of the sensor. The Δ*I* of the humidity sensor decreases from 23.10 to 2.87 μA as the height above the water increases from 2 to 12 mm.

Based on the above results, we designed a non-contact human–machine interaction system, including a signal collecting unit (CEH sensor), a signal processing and control unit (Arduino), and a signal output unit (elevator model). Compared with the conventional contact control system, the non-contact control system can avoid the spread of the virus, as illustrated in Fig. 4d,e. As shown in Fig. 4f, g, we used a conventional touch button and a non-contact human–machine interface based on the humidity sensor to control an elevator model (Supplementary Movie 5), respectively. The non-contact controller based on the humidity sensor can realize the control of an elevator by just approaching the button with a finger without real contact.

Phosphors that can emit fluorescence under ultraviolet (UV) light were used to simulate viruses. After operating the contact controller, the index finger was infected by the viruses (Fig. 4h). In contrast, after operating the non-contact controller, the finger was not infected (Fig. 4i), indicating the non-contact human–machine interaction system based on the CEH sensor can effectively prevent virus transmission and bacterial infection.

## Discussion

In summary, we report a self-powered CEH sensor driven by metal-air redox reaction and demonstrate its high performance in remote health management and medical treatment. The CEH sensor is composed of GO/SF/LiBr gel electrolyte sandwiched between an active anode and an inert cathode, which can be fabricated through a facile printing process using a GO/SF/LiBr dispersion that shows excellent processability. Humidity can be sensitively detected by the sensor by tracking its output current, which depends on the ion mobility of the electrolyte that is highly affected by the content of water. The high sensitivity can be ascribed to the abundant hydrophilic groups in GO and SF as well as the good hygroscopicity of LiBr. Furthermore, the unique layered brick-and-mortar structure of GO/SF/LiBr hinders the fast migration of ions and thus improves the lifetime of the sensor. The sensor shows high sensitivity (0.09 μA/s/1%), short response (1.05 s) and recovery (0.80 s) time, and a wide RH working range (11–84%). For demonstration purposes, we designed a smart respiratory system that can monitor, diagnose, and treat SAS. In addition, we fabricated a non-contact human–machine interface using the CEH sensor, which can effectively block virus transmission and bacterial infection. We anticipate that this strategy of fabricating self-powered sensors based on metal-air redox reactions may not only provide a humidity sensor for health management but also inspire new pathways for designing and fabricating power-free devices for portable and wearable applications.

## Methods

### Materials

Lithium Bromide and sodium bicarbonate were obtained from Meryer. LiCl, MgCl_2_, K_2_CO_3_, NaBr, NaCl, and KCl were purchased from Aladdin. Silk cocoon was purchased from Alibaba. The graphite paper, copper foil, copper wire, and copper mesh were purchased from Tanqianlang New Materials Co., Ltd. All reagents were used directly without further purification. Deionized water was produced through Master-R and used throughout the study.

### Preparation of SF/LiBr Solution

Bombyx mori silkworm cocoons were cut into small pieces and degummed in boiling water with 0.5 wt% NaHCO_3_ for half an hour. 2.0 g of degummed silk fibers were added into 10 ml LiBr solution (9.3 mol/L) to form a SF/LiBr solution at 80 °C for 1 h.

### Preparation of GO/SF/LiBr Ink

Graphene oxide powder was purchased from Suzhou Tanfeng Graphene Technology Co., Ltd., China. To obtain GO/SF/LiBr ink with different viscosities, SF/LiBr solution (prepared in the previous step) and GO (GO content:2.5 –10 wt%) were mixed in a speed mixer at 3500 rpm for 10 min. Unless otherwise stated, the GO content of the GO/SF/LiBr ink used in this work is 7.5 wt%.

### Fabrication process of the custom-designed GO/SF/LiBr electrolytes

GO/SF/LiBr ink with different viscosities can be printed on various electrodes to prepare custom-designed patterns through various techniques, including direct writing, screen printing, and extrusion printing. For the direct writing method, the designed patterns were written on substrates using a Chinese brush with GO/SF/LiBr (GO content: 2.5 wt%) ink. For the screen printing method, the designed patterns were printed on substrates using a stencil mask with a custom-designed pattern with GO/SF/LiBr (GO content: 7.5 wt%) ink. For the extrusion printing, the designed pattern was printed using a 3D printer (Anycubic I3 MEGA). Specifically, the pattern was designed using 3ds Max and then the pattern was converted into G-code through Ultimaker Cura. The GO/SF/LiBr (GO content: 10.0 wt%) ink was loaded in an injection syringe (150 µm in diameter) and was extruded by a syringe pump (Lead Fluid TSD01) at a speed of 200 μL/min, The moving speed of the nozzle was set at 10 mm/s. Unless otherwise stated, the custom-designed GO/SF/LiBr electrolytes were printed through the screen printing method.

### Fabrication process of the CEH sensors based on metal-air redox reaction

The CEH sensor was prepared by assembling GO/SF/LiBr electrolytes, flexible inert electrodes, and metal electrodes. The custom-designed GO/SF/LiBr electrolyte was directly printed on the flexible inert electrode (graphite paper) through the above method. Then, a flexible active metal electrode (aluminum foil or copper foil) was directly attached to the GO/SF/LiBr. Finally, copper wires were attached to the inert electrode and active metal electrode using silver paste for testing and applications. Unless otherwise stated, the CEH sensor used for test and application was prepared with flexible graphite paper and aluminum foil.

### Performance test of the humidity sensor

The electrical measurement was carried out using an electrochemical workstation (CHI760E, CH Instruments, Inc.). During humidity sensing tests, water vapor was introduced into the testing chamber, in which specific RH can be precisely controlled by using different saturated salt solutions. A schematic illustration of the test set is shown in Supplementary Fig 5. Specific RH (11.3%, 32.8%, 43.2%, 57.6%, 75.3%, and 84.3%) conditions were achieved with different saturated salt solutions (LiCl, MgCl_2_, K_2_CO_3_, NaBr, NaCl, and KCl). Unless otherwise stated, the experiments were carried out at 25 °C, 1 atm. During the temperature stability test, the temperature range was 0–80 °C. During the test of the effect of air pressure and O_2_ concentration, the sensor was placed in a vacuum chamber. The air pressure was pumped from 1 atm to 0.01 atm and held for 30 min. During the lifetime test, the RH was controlled at 25%. The tests were repeated three times independently with similar results. During non-contact sensing tests, the distances of the humidity sensor above the water (37 °C) were controlled using a SHIMADZUAG-IS tester. Data were analyzed through Origin 2018 edu.

### Characterizations

The topography of GO flake and GO/SF/LiBr composite was characterized using an AFM (Cypher VRS, Oxford Instruments.) and Cryo-SEM (Quanta 450, FEI). Each experiment was repeated three times independently and produced similar results. The rheological properties of the GO/SF/LiBr inks were measured by a rheometer (MCR 301; Anton Paar). The viscosities of the ink were recorded within a shear rate from 10^−1^ to 10^3^ s^−1^. The thermal stability and water content (11.3% RH) of the GO/SF/LiBr ink were characterized using a thermogravimetric analyzer (Ar, 10 °C/min, Q5000IR, TA Instruments). Raman spectra of the GO were collected using a Raman spectroscope (Horiba HR800, 532 nm). X-ray photoelectron spectra (XPS) of graphite paper and GO were collected using a PHI Quantera SXM (ULVAC-PHI) scanning X-ray photoelectron spectrometer microprobe.

### Demonstration of humidity sensor on telemedicine

A CEH sensor was integrated into the mask. The respiratory data were collected through Arduino, and then the data were sent to the cloud (bemfa.com) through the ESP8232 Wi-Fi module. The respiratory data were observed in real-time through the mobile phone (iPhone Xs) at the website (bemfa.com) and further used a Python program to read cloud data and displayed it on a laptop (Lenovo X1 Carbon 2017). Consent to publish identifiable images of research participants was obtained.

### Demonstration of humidity sensor on respiratory monitoring

A CEH sensor was integrated into the mask. The respiratory data were collected through Arduino, and real-time processing and judgment were performed through an Arduino program at the same time. According to the judgment result, the LED, buzzer, and air pump were further controlled with Arduino. Two healthy subjects, including 1 male and 1 female, were involved in the respiratory monitoring test. Consent to publish identifiable images of research participants was obtained.

### Demonstration of humidity sensor on non-contact human–machine interaction

The humidity change signal of the finger approached were collected by Arduino and a CEH sensor, and real-time processing and judgment were performed with an Arduino program at the same time. The motor (elevator) was controlled according to the judgment result.

### Ethics declarations

The data were obtained with the informed consent of all participants. The Institutional Review Board of Tsinghua University approved this study (NO. 20220118).

### Reporting summary

Further information on research design is available in the Nature Research Reporting Summary linked to this article.

## Supplementary information

Supplementary Information

Description of Additional Supplementary Files

Supplementary Movie 1

Supplementary Movie 2

Supplementary Movie 3

Supplementary Movie 4

Supplementary Movie 5

Reporting Summary

## Data Availability

The data generated in this study have been deposited in the Science Data Banke database under accession code http://cstr.cn/31253.11.sciencedb.01874^59^.

## Code availability

The source codes for apnea diagnosis, recognition, treatment, and codes for telemedicine and human–machine interaction used in this study are available at https://github.com/LSTHU/Respiratory_sensing^60^.
