# Supplementary material for: Humidity-sensitive chemoelectric flexible sensors based on metal-air redox reaction for health management
Source: Nat Commun. 2022 Sep 15;13:5416. doi: 10.1038/s41467-022-33133-y (PMC9477177; doi:10.1038/s41467-022-33133-y)
Supplement: Supplementary file 1 — Supplementary Information [file 41467_2022_33133_MOESM1_ESM.pdf]

Supplementary Information

Humidity-sensitive chemoelectric flexible sensors based on metal-air redox reaction for health management

*Shuo Li, Yong Zhang, Xiaoping Liang, Haomin Wang, Haojie Lu, Mengjia Zhu, Huimin Wang, Mingchao Zhang, Xinping Qiu, Yafeng Song, and Yingying Zhang**

The supplementary file includes:

Supplementary Method: Oxygen Reduction Reaction (ORR) Activity of the Graphite Paper

Supplementary Note 1: Theoretical analysis on the influence of temperature, air pressure and O_2 pressure on the sensor

Supplementary Figure 1: X-ray photoelectron spectrum (XPS) of graphene oxide (GO). (a) Fule XPS and element content of GO. (b) High-resolution C *1s* XPS of GO.

Supplementary Figure 2: Photo images of silk fiborin (SF)/LiBr and graphene oxide (GO)/SF/LiBr ink, showing the uniform dispersion of GO in SF.

Supplementary Figure 3: Transmission electron microscopy images (TEM), showing the silk fibroin (SF)/LiBr adsorbed on the graphene oxide (GO) sheet. (a) TEM of GO. (b) TEM of GO/SF/LiBr composites. The experiment was repeated 3 times independently with similar results.

Supplementary Figure 4: Atomic force microscopy image of pure graphene oxide (GO). (a) Morphology of pure GO flakes. (b) Profile of pure GO flakes. The experiment was repeated 3 times independently with similar results.

Supplementary Figure 5: Intensity ratio of D band to G band (I_D/I_G) of the Raman spectra of graphene oxide (GO) and GO/silk fibroin (SF)/LiBr composites, indicating the absorption of SF/LiBr on GO.

Supplementary Figure 6: Viscosities of silk fibroin (SF)/graphene oxide (GO) inks with different GO contents at different shear rates.

Supplementary Figure 7: Schematic illustration and photo images of the various preparation process of chemoelectric humidity sensors based on graphen oxide (GO)/silk fibroin (SF)/LiBr ink with different viscosities. (a) Direct printing. (b) Screen printing. (c) Extrusion printing.

Supplementary Figure 8: Photos of the deformed chemoelectric humidity sensors, showing the flexibility.

Supplementary Figure 9: Cryo-scanning electron microscopy image of the cross-section of a graphene oxide (GO)/silk fibroin (SF)/LiBr film. The experiment was repeated 3 times independently with similar results.

Supplementary Figure 10: Open-circuit voltage of a chemoelectric humidity sensor under different environment. (a) Absolute dry environment.(b) Ambient (30% relative humidity (RH)) environment.

Supplementary Figure 11: Oxygen reduction reaction activity characterization. (a) Cyclic voltammograms of graphite in N_2 and O_2 saturated 0.1 M KOH solution. (b) Linear sweep voltammetry polarization curve of graphite in O_2 saturated 0.1 M KOH solution.

Supplementary Figure 12: X-ray photoelectron spectrum of graphite paper.

Supplementary Figure 13: Schematic illustration of the humidity testing set.

Supplementary Figure 14: Sensitivity of the sensor. Data are presented as mean values ± SD. n = 3 independent experiments.

Supplementary Figure 15: Short-circuit current change (ΔI) of the chemoelectric humidity sensor under different relative humidity (RH). Data are presented as mean values ± SD. n = 3 independent experiments.

Supplementary Figure 16: Influence of temperature on the humidity sensor. (a) The dependence of open-circuit voltage of the humidity sensor on the temperature. (b) The response of the chemoelectric humidity sensor to humidity changes (relative humidity (RH) 0%–43.2%) at different temperature. (c) The dependence of current change rate ($\mathrm{d}I/\mathrm{d}t$) on temperature when the RH changes from 0% to 44%. Data are presented as mean values ± SD. n = 3 independent experiments.

Supplementary Figure 17: Dependence of open-circuit voltage of the humidity sensor on the air pressure and O_2 pressure (air pressure is pumped from 1 atm to 0.01 atm).

Supplementary Figure 18: Influence of the type of anode on the chemoelectric humidity sensor. (a) Dependence of open-circuit voltage on anode materials and structures. (b) Response to humidity changes (RH 0%–43.2%) of the chemoelectric humidity sensor using Cu foil as the anode.

Supplementary Figure 19: Anode consumption of the chemoelectric humidity sensors after 12 hours of works. The active area of the anode is 2.0 × 2.0 cm^2.

Supplementary Figure 20: Open circuit voltage of the humidity sensor over time. Data are presented as mean values ± SD. n = 3 independent experiments.

Supplementary Figure 21: Response of the sensor to switch of relative humidity (RH) between 0% and 43.2% when the sensor has continuously worked for different duration, showing its stability over time. (a) Response of the sensor worked for 0 hours. (b) Response of the sensor worked for 12 hours. (c) Response of the sensor worked for 24 hours. (d) Response of the sensor worked for 36 hours. (e) Response of the sensor worked for 48 hours. (f) Response of the sensor worked for 60 hours. (g) Response of the sensor worked for 72 hours. (h) Response of the regenerated sensor.

Supplementary Figure 22: Response of the chemoelectric humidity sensor to humidity changes (relative humidity (RH) 0%–43.2%) after 180 days of storage.

Supplementary Figure 23: Load-deformation curve of the chemoelectric humidity sensor during 300 bending cycles (Sample size: 80 mm × 10 mm).

Supplementary Figure 24: Response of the chemoelectric humidity sensor to humidity changes (relative humidity (RH) 0%–43.2%) before and after bending. (a) Response of the sensor before bending. (b) Response of the sensor after 300 bending cycles.

Supplementary Figure 25: Water contact angle of graphene oxide (GO)/silk fibroin (SF)/LiBr surface, indicating excellent hydrophilicity of the GO/SF/LiBr electrolyte.

Supplementary Figure 26: Water absorption content and relative resistance change of graphene oxide/silk fibroin/LiBr membranes (10 mm × 10 mm × 0.1 mm) under different relative humidity. (a) Water absorption content. (b) Relative resistance change. Data are presented as mean values ± SD. n = 3 independent experiments.

Supplementary Figure 27: Thermogravimetric analysis of graphene oxide (GO)/silk fibroin (SF)/LiBr composites.

Supplementary Figure 28: Signals of different breathing states detected by the chemoelectric humidity sensors. For the inhalation process, the current change rate of the sensor is negative due to the lower relative humidity of the ambient air. In contrast, for the exhalation process, the current change rate of the humidity sensor is positive due to the higher relative humidity of the exhaled air. (a) Fast breathing. (b) Normal breathing (Blue: Inhale, Red: Exhale).

Supplementary Figure 29: Structure of respiratory monitoring-diagnosing-treatment systems. (a) A respiratory monitoring and telemedicine system. (b) A sleep apnea syndrome (SAS) diagnosis and treatment system which can alert people with SAS by buzzer and light-emitting diode (LED). (c) A SAS diagnosis and treatment system that controls ventilator in time by monitoring the breathing status.

Supplementary Figure 30: Photo of the readout electronics.

Supplementary Figure 31: Dependence of humidity on distance from the index finger surface. Temperature: 25 °C; relative humidity (RH): 48%

Supplementary Table 1: Comparison of the response time and recovery time of the our chemoelctric sensors and other reported flexible humidity sensors.

References 1-20

Supplementary Method: Oxygen Reduction Reaction (ORR) Activity of the Graphite Paper

The oxygen reduction reaction activity was characterized using a three-electrode cell (working electrode: rotating disk electrode (RDE-E5), counter electrode: platinum wire, reference electrode: saturated Ag/AgCl). The signals were recorded with an electrochemical workstation (CHI 760E), then the potentials were calibrated to the RHE potential according to the equation $E_{RHE} = E_{Ag/AgCl} + (0.197 + 0.0591\ pH)$ V. 3 mg expandable graphite powder was dispersed into 1 mL ethanol/Nafion (1.85 wt%) mixed solution by ultrasonication (30 min) to form a homogenous ink. 10 μL ink was dropped onto the rotating disk electrode and then naturally dried in air. Cyclic voltammograms were recorded at 50 mV/s in N_2 and O_2 saturated 0.1 M KOH. The linear sweep voltammetry polarization curve for ORR was characterized at 5 mV/s in O_2 saturated 0.1 M KOH, under the condition of 1600 rpm rotation rate and 95% iR-compensation.

Supplementary Note 1: Theoretical analysis on the influence of temperature, air pressure and O_2 pressure on the sensor

For the self-powered chemoelectric humidity (CEH) sensor, oxidation and reduction reactions occur on the anode and cathode surfaces, respectively, which can be expressed as the following:

$$\frac{1}{n}M \rightarrow \frac{1}{n}M^{n+} + e^- \quad (1)$$

$$\frac{1}{4}O_2 + e^- + \frac{1}{2}H_2O \rightarrow OH^- \quad (2)$$

where M stands for the active metal in the anode.

Based on the Nernst equation, the electrode potential of anode and cathode can be calculated as:

$$\varphi_{O_2|OH^-} = \varphi^{\Theta}_{O_2|OH^-} - \frac{RT}{F}\ln\frac{a_{OH^-}}{(p_{O_2}/p^{\Theta})^{1/4}} \quad (3)$$

$$\varphi_{M^{n+}|M} = \varphi^{\Theta}_{M^{n+}|M} - \frac{RT}{F}\ln\frac{1}{a_{M^{n+}}{}^{1/n}} \quad (4)$$

where $\varphi^{\Theta}_{O_2|OH^-}$ and $\varphi^{\Theta}_{M^{n+}|M}$ is the standard redox potential, and the a_{OH^-}, $a_{M^{n+}}$, and p_{O_2} denotes the ionic activity and oxygen pressure on the electrode surface.

then, the cell potential of the metal-air battery can be calculated as:

$$E = E^{\Theta} - \frac{RT}{F}\ln\frac{a_{OH^-} * a_{M^{n+}}{}^{1/n}}{(p_{O_2}/p^{\Theta})^{1/4}} \quad (5)$$

where E^{Θ} is the standard cell potential.

The dependence of potential on the temperature can be expressed as:

$$\frac{\partial E}{\partial T} = -\frac{R}{F}\ln\frac{a_{OH^-} * a_{M^{n+}}{}^{1/n}}{(p_{O_2}/p^{\Theta})^{1/4}} \quad (6)$$

Since $a_{OH^-} * a_{M^{n+}}$ are very small (below 10^{-11}, according to the solubility product of $Al(OH)_3$) in our system,

$$\frac{\partial E}{\partial T} > 0 \quad (7)$$

Therefore, the cell potential of the metal-air battery increases with temperature.

On the other side, the dependence of cell potential on the oxygen pressure can be expressed as:

$$\frac{\partial E}{\partial p_{O_2}} = \frac{RT}{4F} * \frac{1}{p_{O_2}} \quad (8)$$

If the temperature is 298.15 K, p_{O_2} is 200 Pa (corresponding to air pressure of 0.01 atm),

$$\frac{\partial E}{\partial p_{O_2}} \approx 3.2*10^{-5}\ \text{V/Pa} \quad (9)$$

indicating the air pressure and O_2 pressure has an ignorable influence on the open-circuit voltage of the CEH sensor, which is consistent with the experiment results.

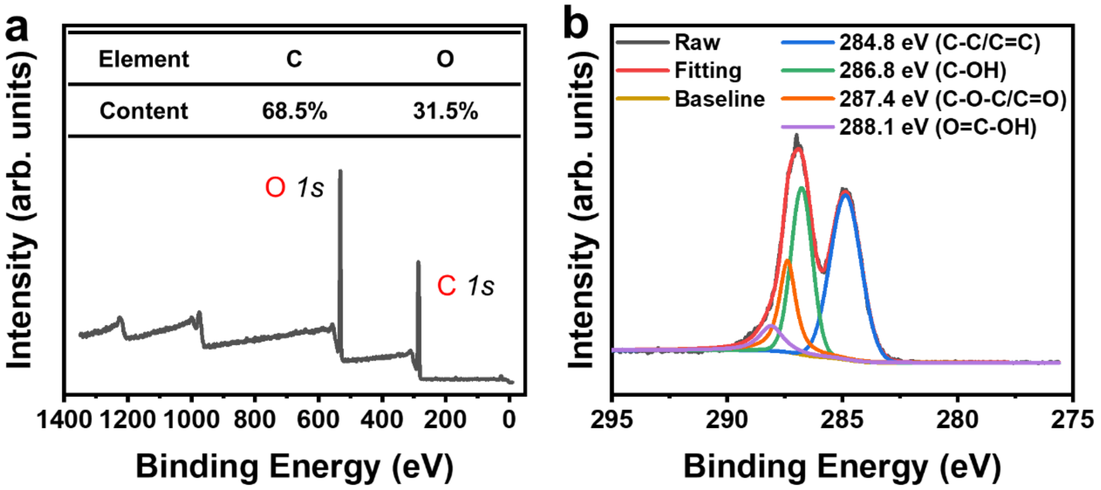

Supplementary Figure 1: **X-ray photoelectron spectrum (XPS) of graphene oxide (GO).** (a) Fule XPS and element content of GO. (b) High-resolution C *1s* XPS of GO.

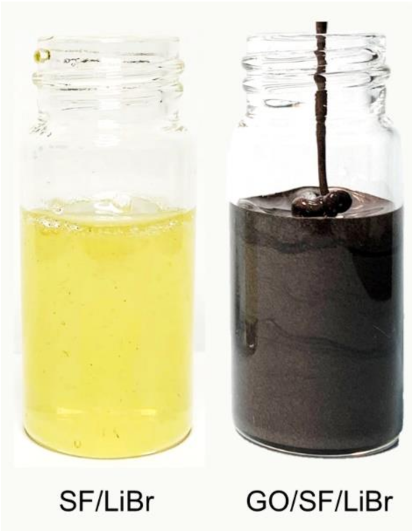

Supplementary Figure 2: Photo images of silk fiborin (SF)/LiBr and graphene oxide (GO)/SF/LiBr ink, showing the uniform dispersion of GO in SF.

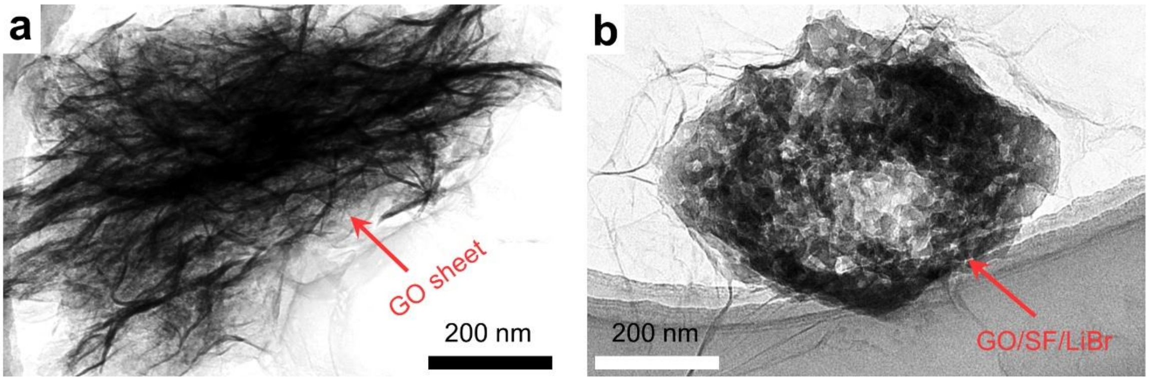

Supplementary Figure 3: Transmission electron microscopy images (TEM), showing the silk fibroin (SF)/LiBr adsorbed on the graphene oxide (GO) sheet. (a) TEM of GO. (b) TEM of GO/SF/LiBr composites. The experiment was repeated 3 times independently with similar results.

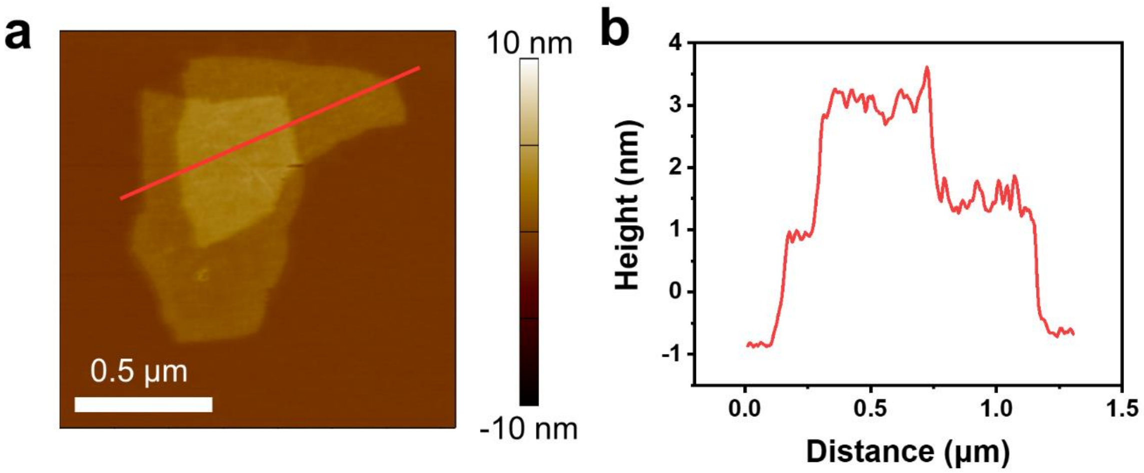

Supplementary Figure 4: Atomic force microscopy image of pure graphene oxide (GO). (a) Morphology of pure GO flakes. (b) Profile of pure GO flakes. The experiment was repeated 3 times independently with similar results.

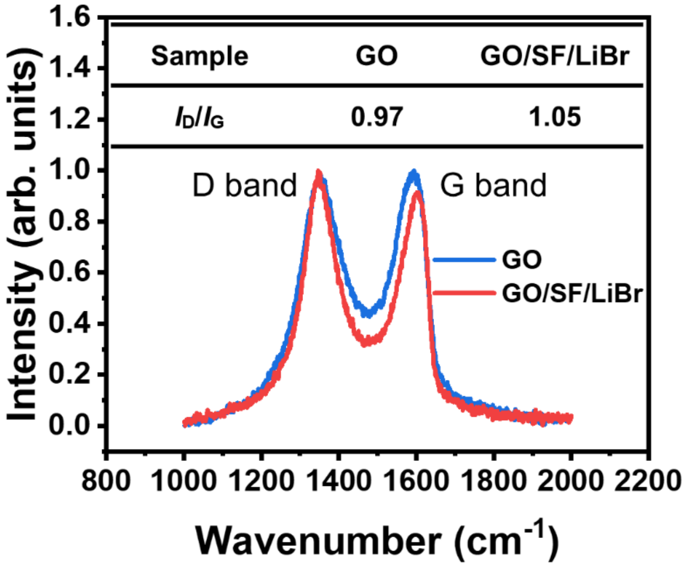

Supplementary Figure 5: Intensity ratio of D band to G band (I_D/I_G) of the Raman spectra of graphene oxide (GO) and GO/silk fibroin (SF)/LiBr composites, indicating the absorption of SF/LiBr on GO.

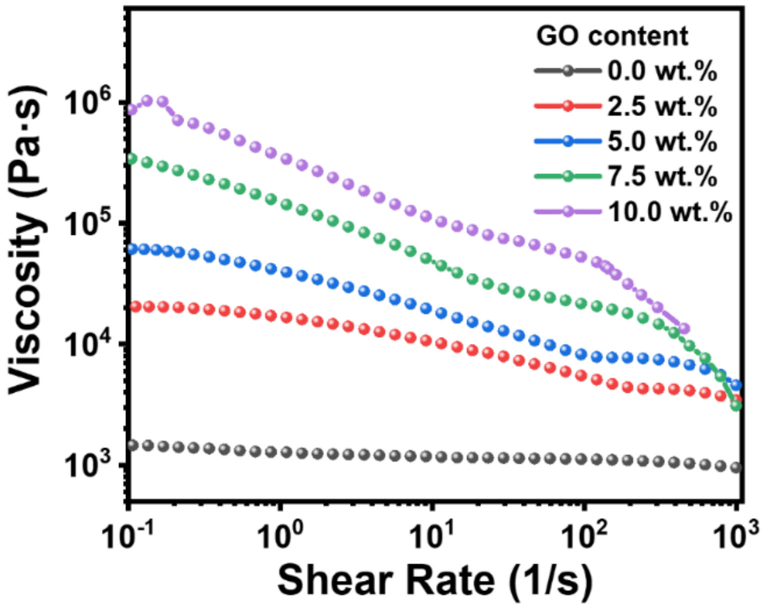

Supplementary Figure 6: Viscosities of silk fibroin (SF)/graphene oxide (GO) inks with different GO contents at different shear rates.

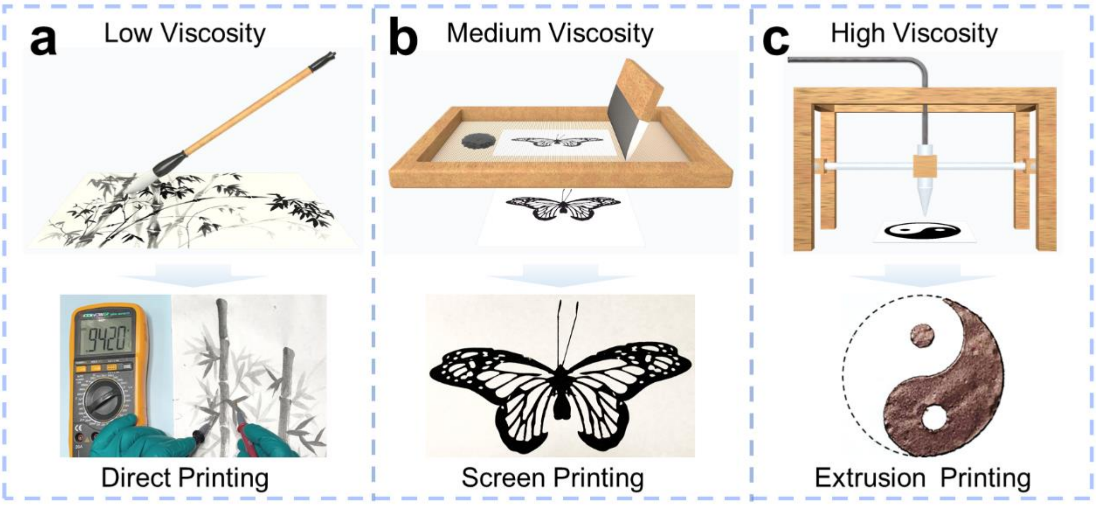

Supplementary Figure 7: Schematic illustration and photo images of the various preparation process of chemoelectric humidity sensors based on graphen oxide (GO)/silk fibroin (SF)/LiBr ink with different viscosities. (a) Direct printing. (b) Screen printing. (c) Extrusion printing.

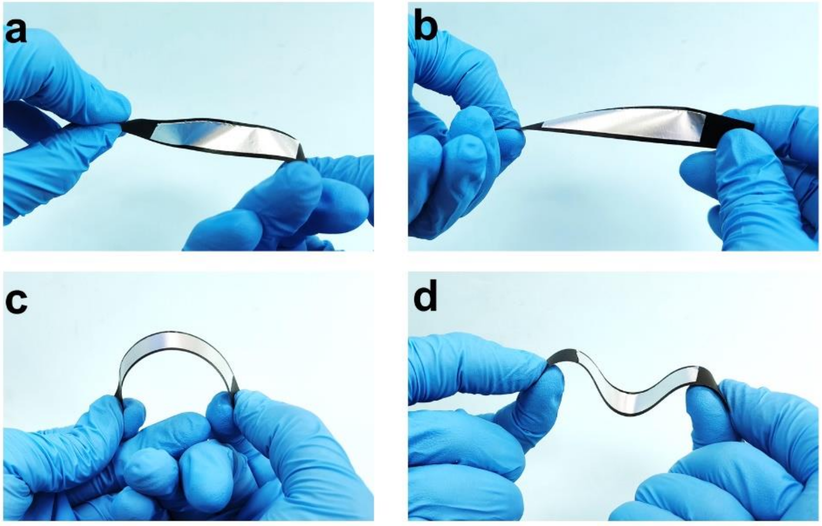

Supplementary Figure 8: Photos of the deformed chemoelectric humidity sensors, showing the flexibility.

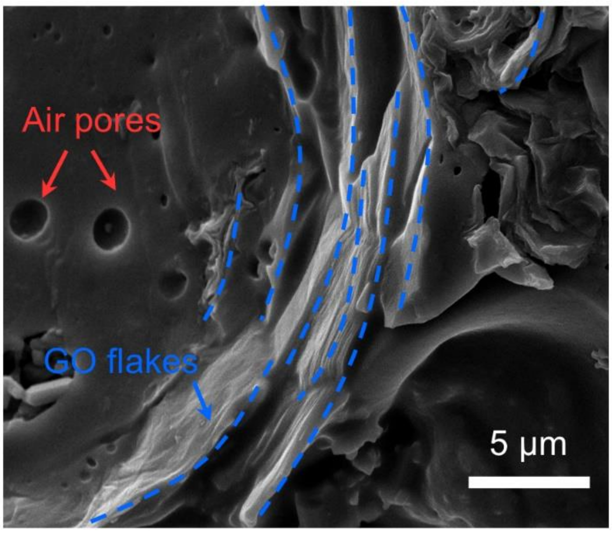

Supplementary Figure 9: Cryo-scanning electron microscopy image of the cross-section of a graphene oxide (GO)/silk fibroin (SF)/LiBr film. The experiment was repeated 3 times independently with similar results.

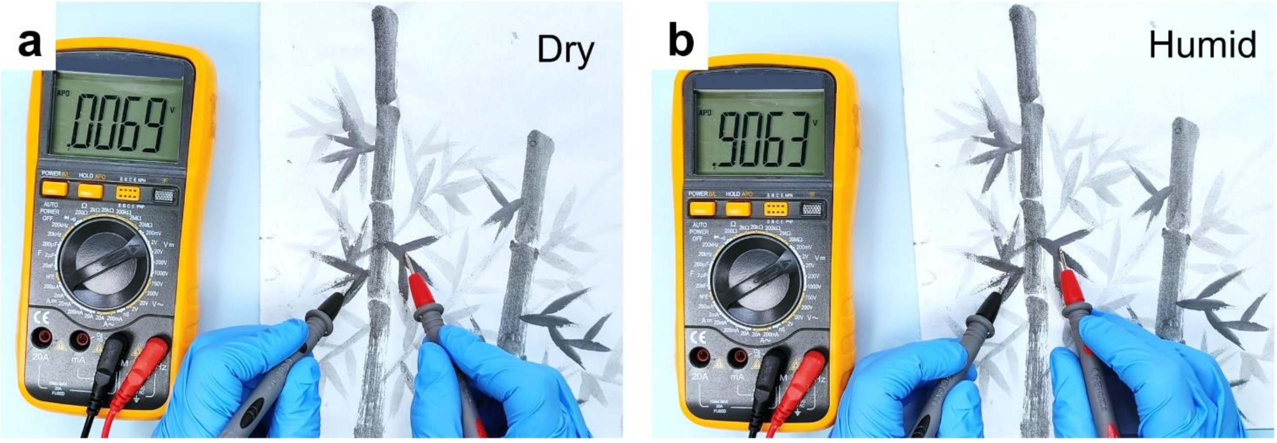

Supplementary Figure 10: Open-circuit voltage of a chemoelectric humidity sensor under different environment. (a) Absolute dry environment.(b) Ambient (30% relative humidity (RH)) environment.

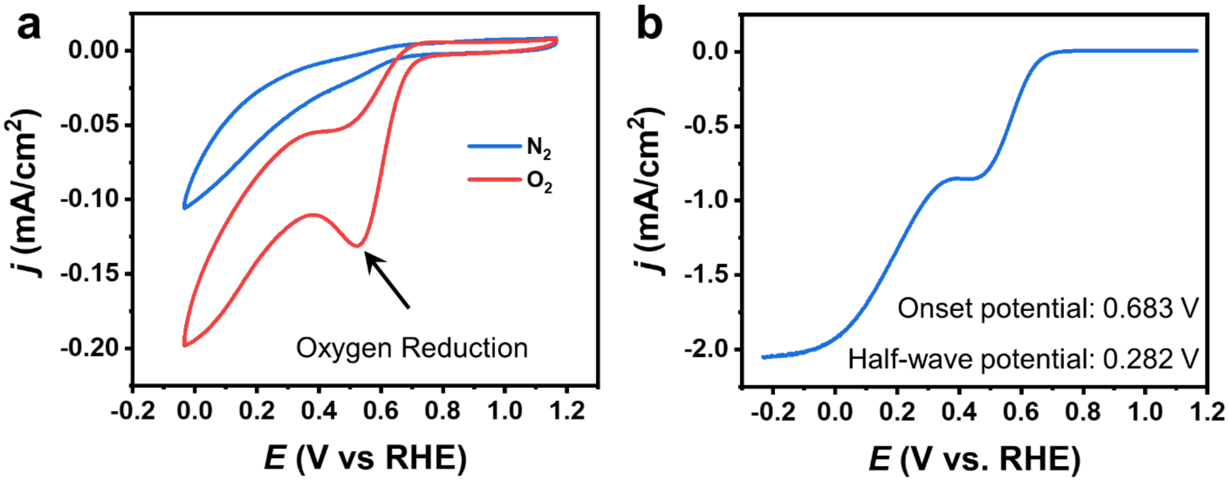

Supplementary Figure 11: Oxygen reduction reaction activity characterization. (a) Cyclic voltammograms of graphite in N_2 and O_2 saturated 0.1 M KOH solution. (b) Linear sweep voltammetry polarization curve of graphite in O_2 saturated 0.1 M KOH solution.

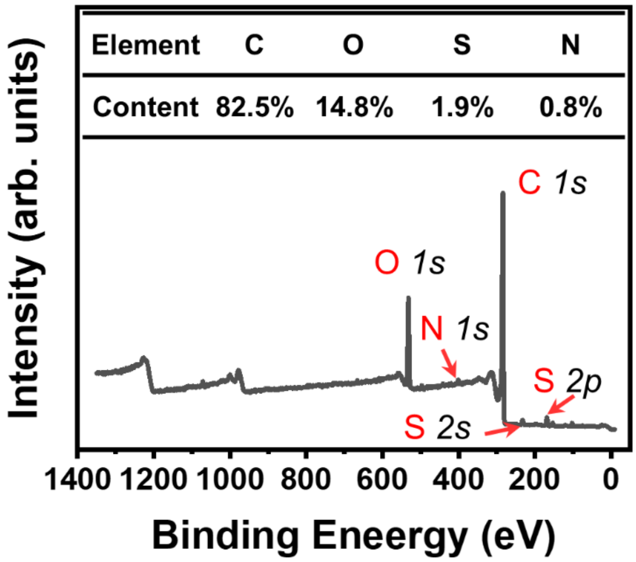

Supplementary Figure 12: X-ray photoelectron spectrum of graphite paper.

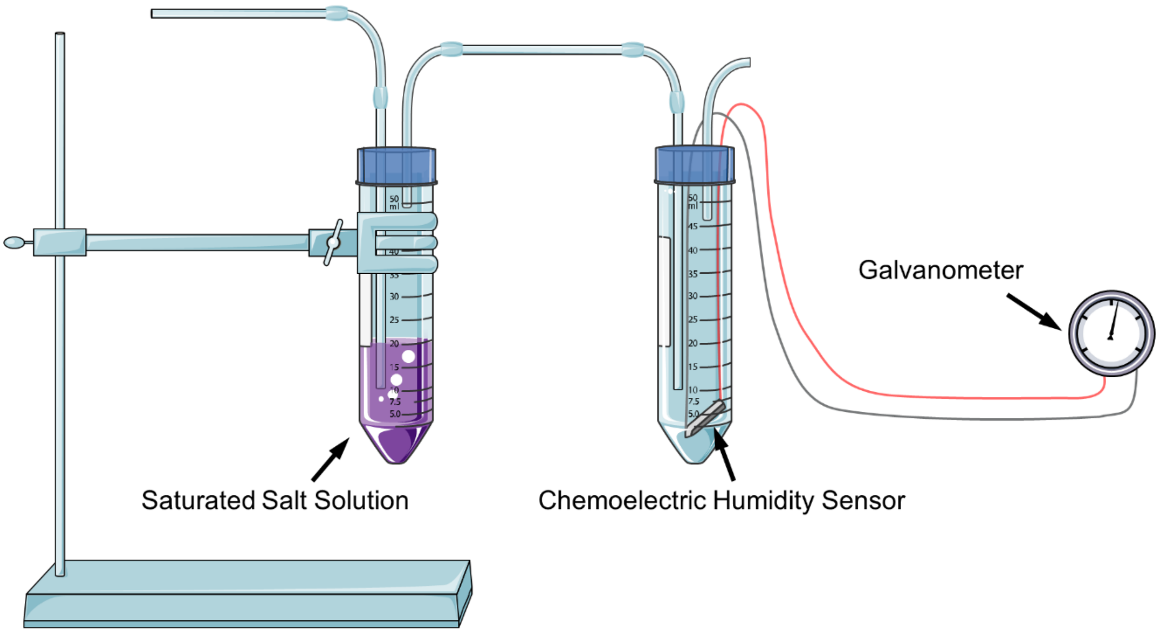

Supplementary Figure 13: Schematic illustration of the humidity testing set.

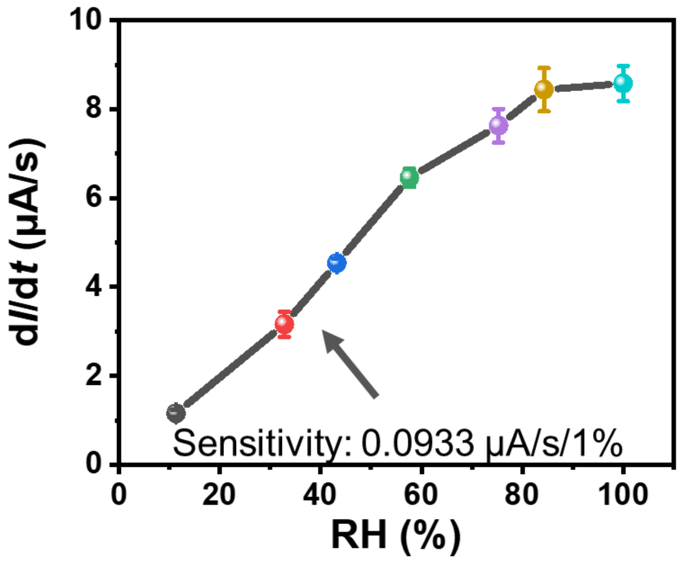

Supplementary Figure 14: Sensitivity of the sensor. Data are presented as mean values ± SD. n = 3 independent experiments.

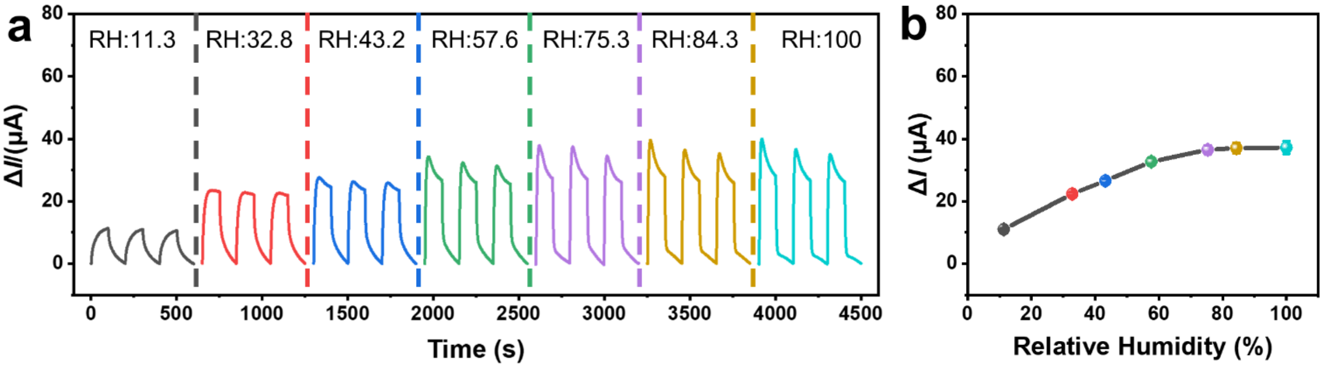

Supplementary Figure 15: Short-circuit current change (Δ*I*) of the chemoelectric humidity sensor under different relative humidity (RH). Data are presented as mean values ± SD. n = 3 independent experiments.

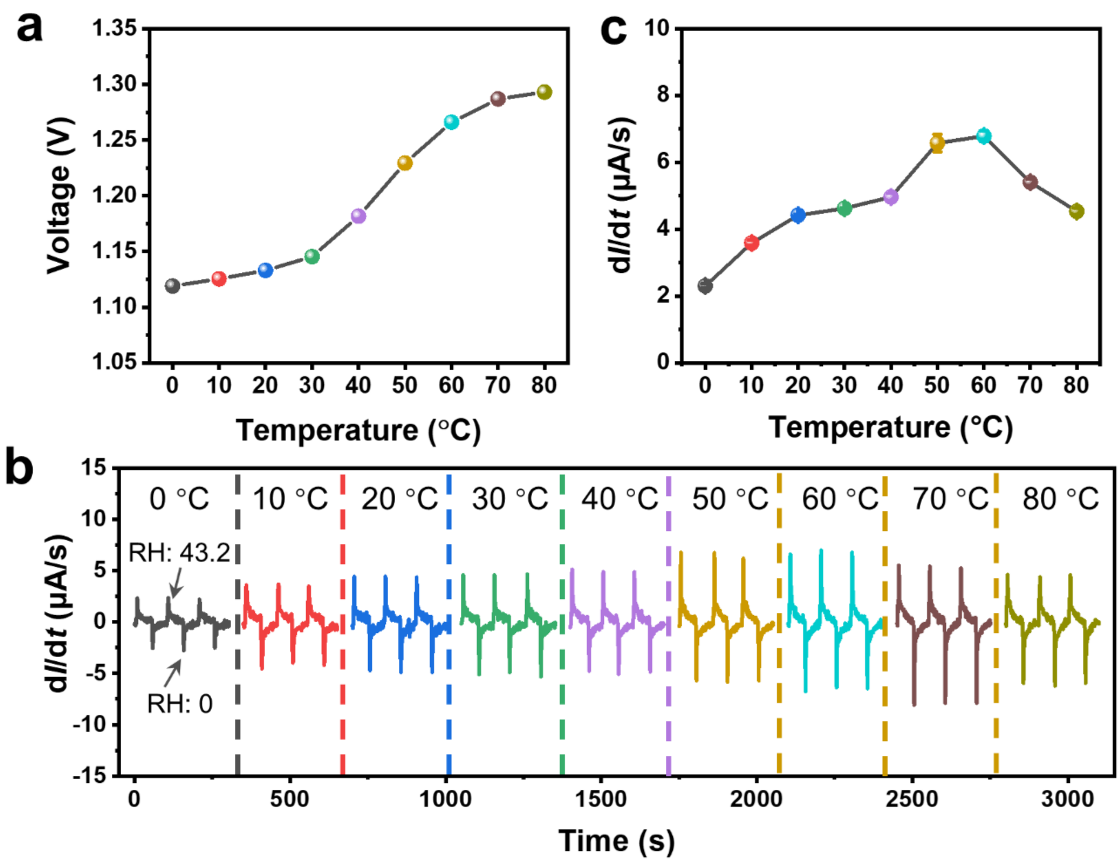

Supplementary Figure 16: Influence of temperature on the humidity sensor. (a) The dependence of open-circuit voltage of the humidity sensor on the temperature. (b) The response of the chemoelectric humidity sensor to humidity changes (relative humidity (RH) 0%–43.2%) at different temperature. (c) The dependence of current change rate (d*I*/d*t*) on temperature when the RH changes from 0% to 44%. Data are presented as mean values ± SD. n = 3 independent experiments.

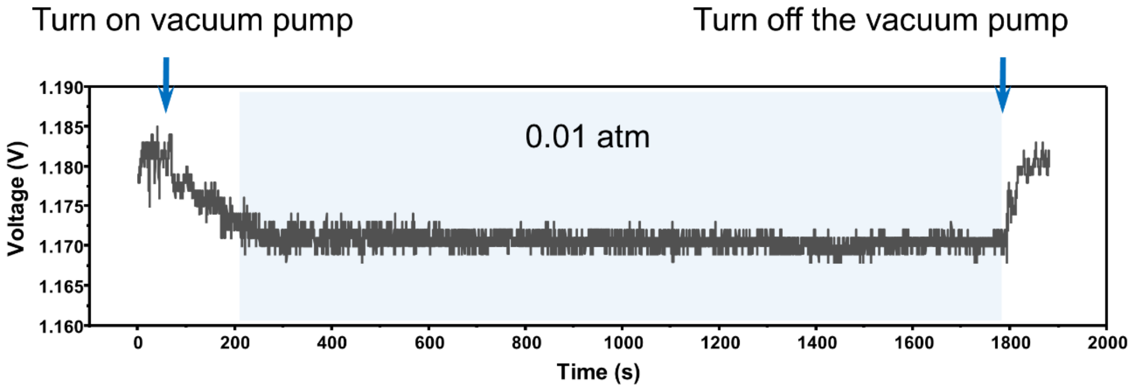

Supplementary Figure 17: Dependence of open-circuit voltage of the humidity sensor on the air pressure and O_2 pressure (air pressure is pumped from 1 atm to 0.01 atm).

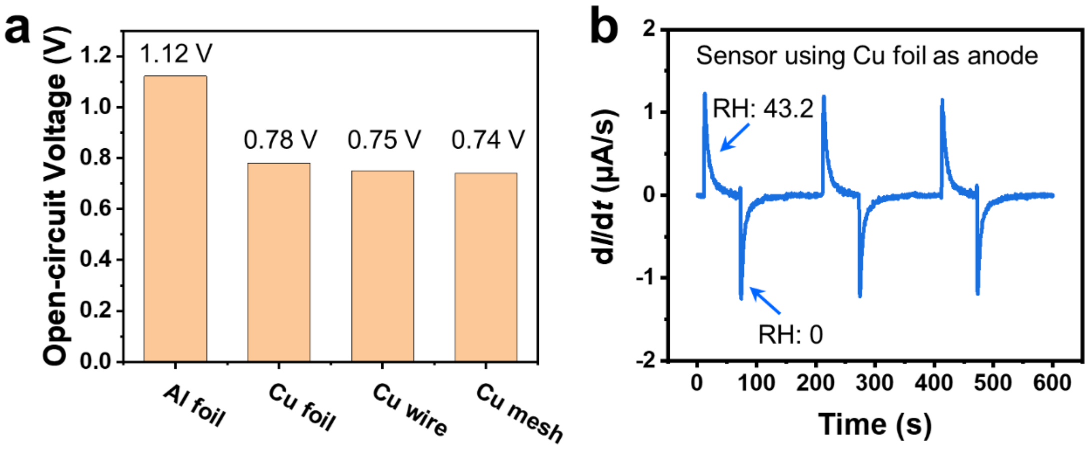

Supplementary Figure 18: Influence of the type of anode on the chemoelectric humidity sensor. (a) Dependence of open-circuit voltage on anode materials and structures. (b) Response to humidity changes (RH 0%–43.2%) of the chemoelectric humidity sensor using Cu foil as the anode.

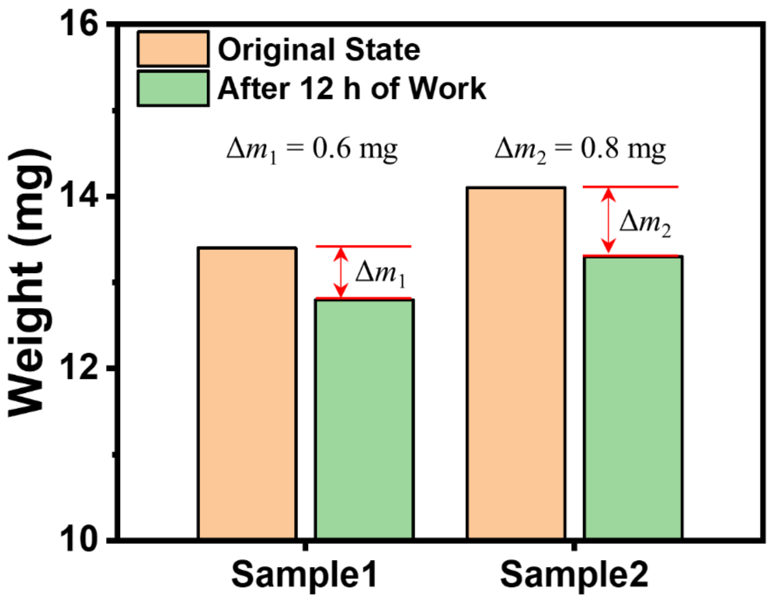

Supplementary Figure 19: Anode consumption of the chemoelectric humidity sensors after 12 hours of works. The active area of the anode is 2.0 × 2.0 cm^2.

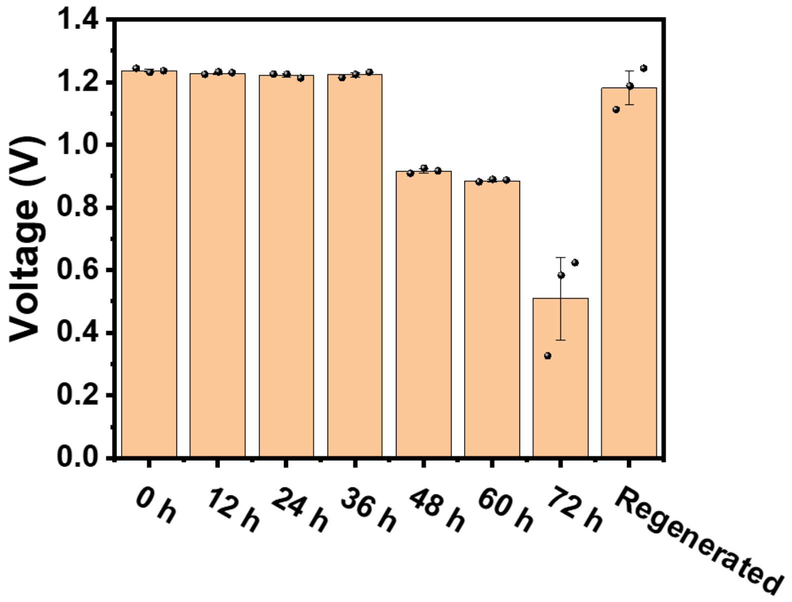

Supplementary Figure 20: Open circuit voltage of the humidity sensor over time. Data are presented as mean values ± SD. n = 3 independent experiments.

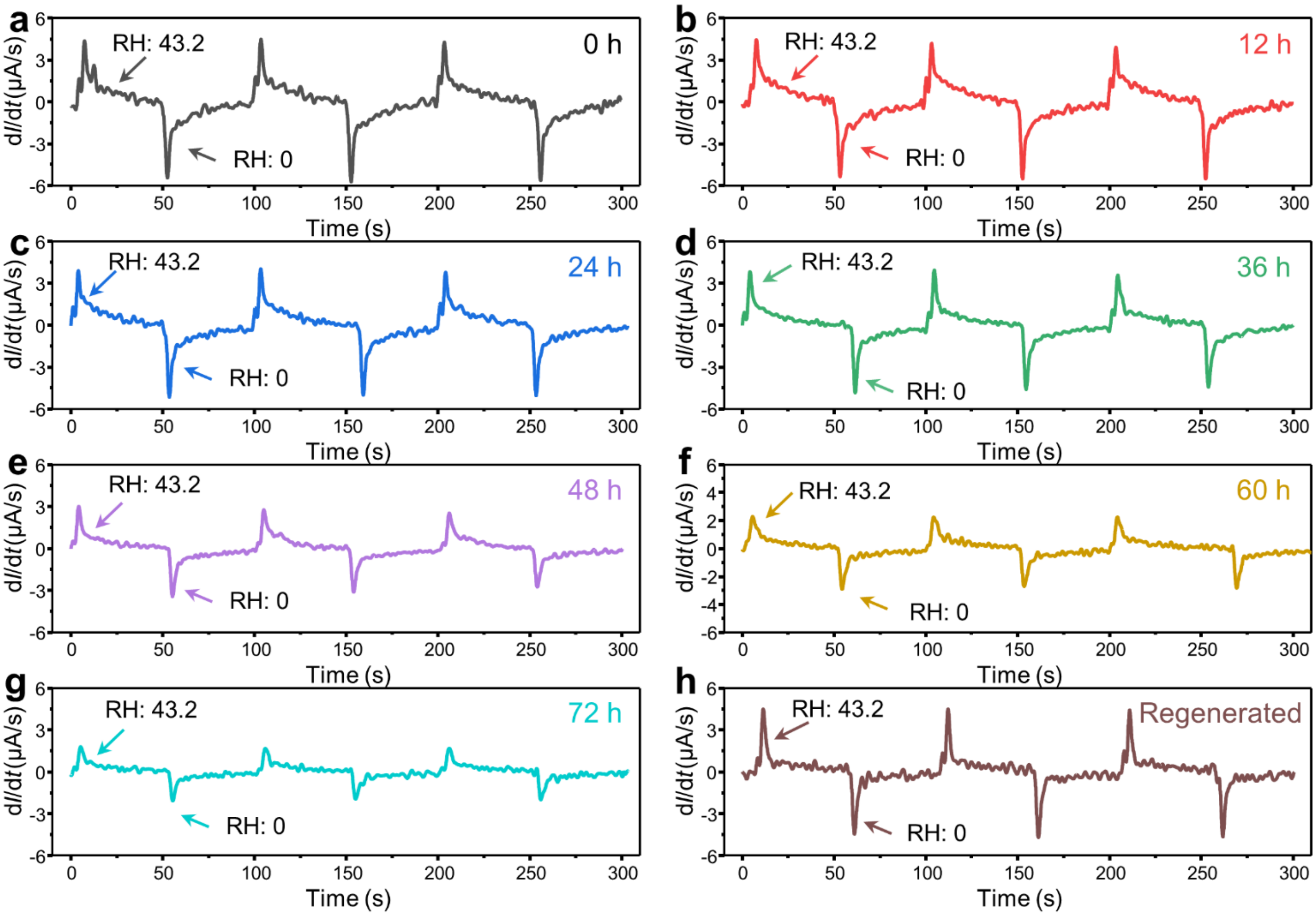

Supplementary Figure 21: Response of the sensor to switch of relative humidity (RH) between 0% and 43.2% when the sensor has continuously worked for different duration, showing its stability over time. (a) Response of the sensor worked for 0 hours. (b) Response of the sensor worked for 12 hours. (c) Response of the sensor worked for 24 hours. (d) Response of the sensor worked for 36 hours. (e) Response of the sensor worked for 48 hours. (f) Response of the sensor worked for 60 hours. (g) Response of the sensor worked for 72 hours. (h) Response of the regenerated sensor.

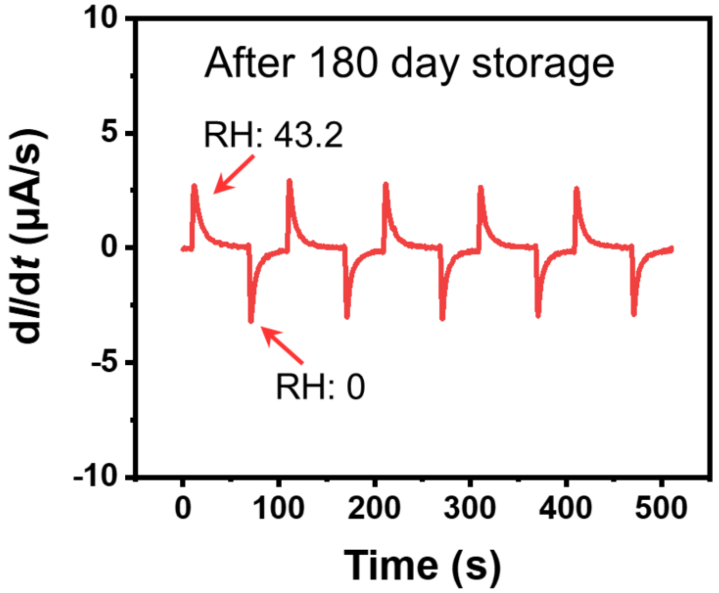

Supplementary Figure 22: Response of the chemoelectric humidity sensor to humidity changes (relative humidity (RH) 0%–43.2%) after 180 days of storage.

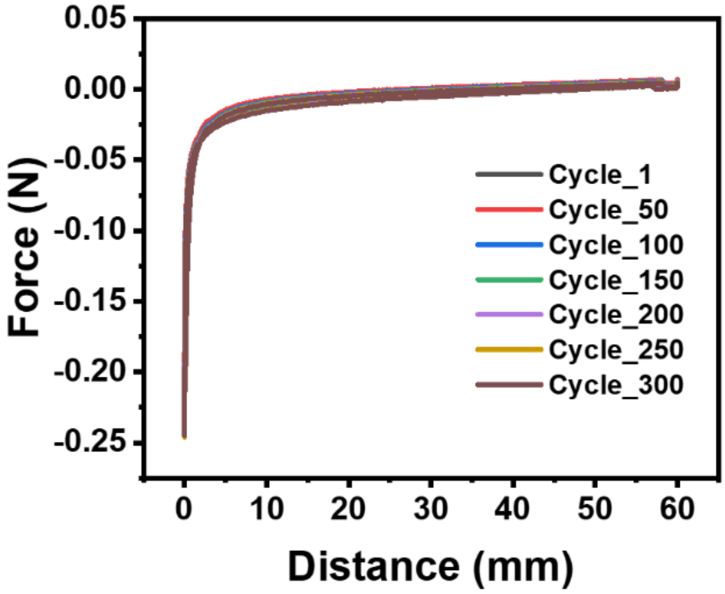

Supplementary Figure 23: Load-deformation curve of the chemoelectric humidity sensor during 300 bending cycles. Sample size: 80 mm×10 mm.

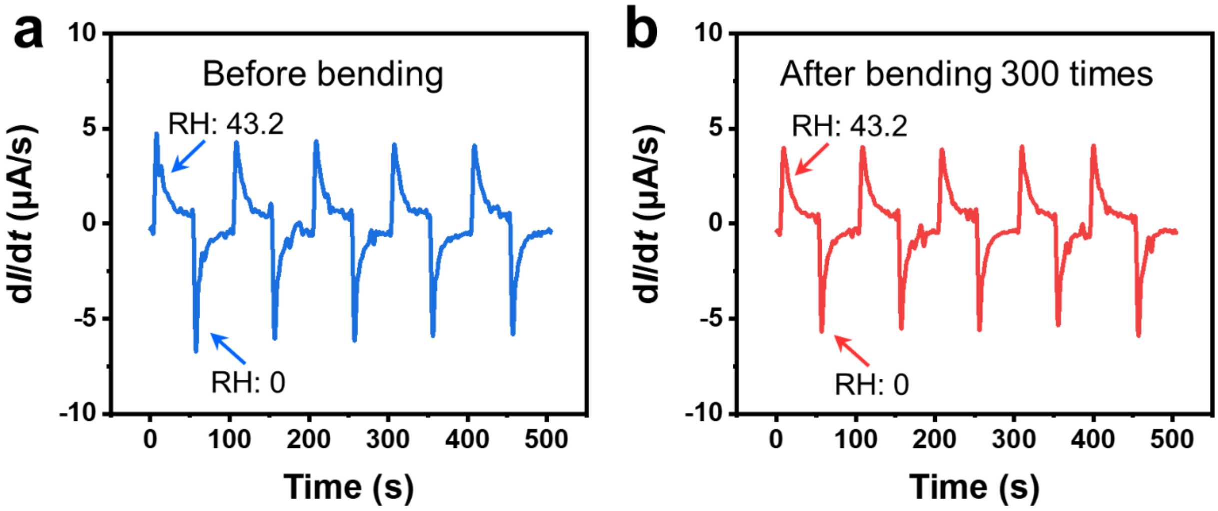

Supplementary Figure 24: Response of the chemoelectric humidity sensor to humidity changes (relative humidity (RH) 0%–43.2%) before and after bending. (a) Response of the sensor before bending. (b) Response of the sensor after 300 bending cycles.

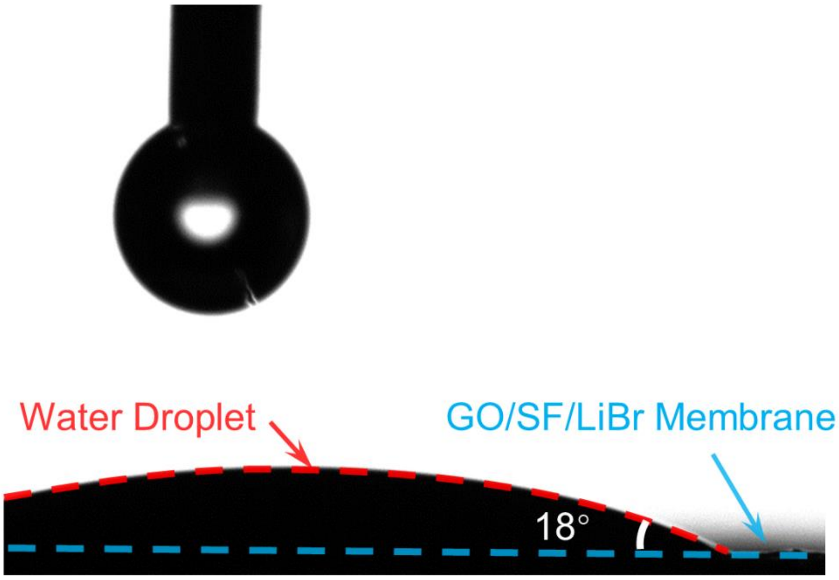

Supplementary Figure 25: Water contact angle of graphene oxide (GO)/silk fibroin (SF)/LiBr surface, indicating excellent hydrophilicity of the GO/SF/LiBr electrolyte.

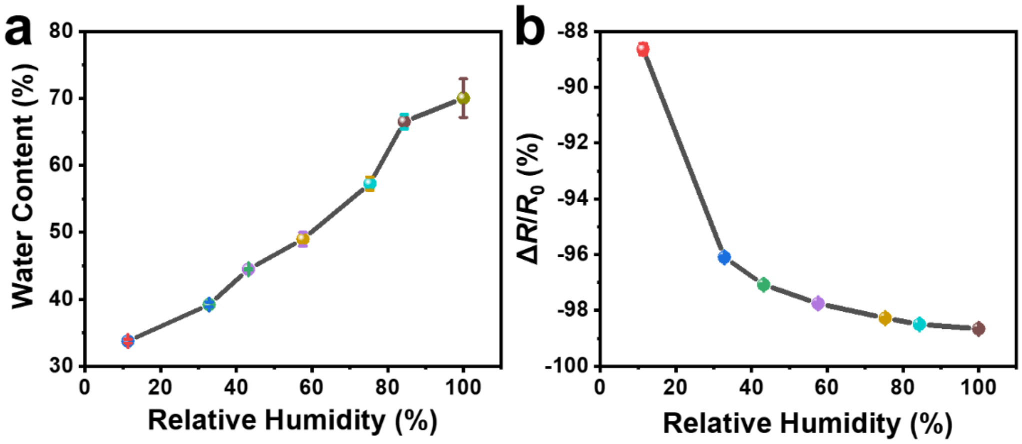

Supplementary Figure 26: Water absorption content and relative resistance change of graphene oxide/silk fibroin/LiBr membranes (10 mm×10 mm×0.1 mm) under different relative humidity. (a) Water absorption content. (b) Relative resistance change. Data are presented as mean values ± SD. n = 3 independent experiments.

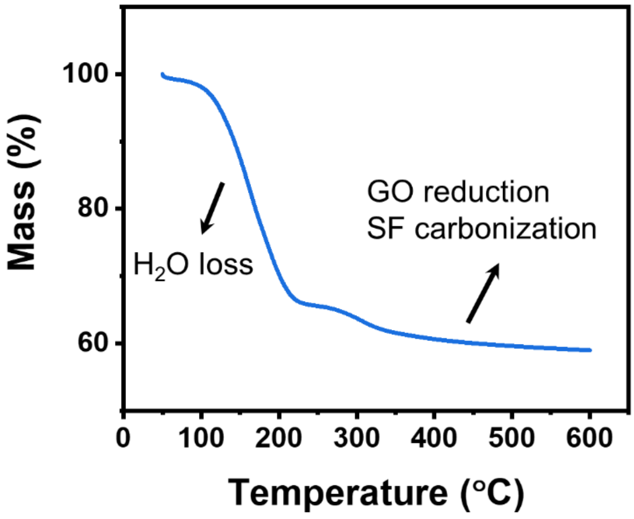

Supplementary Figure 27: Thermogravimetric analysis of graphene oxide (GO)/silk fibroin (SF)/LiBr composites.

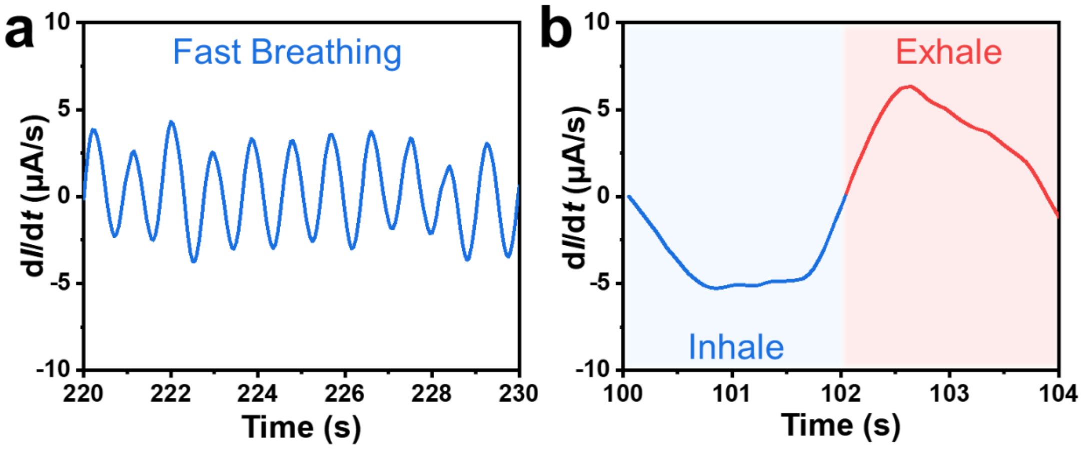

Supplementary Figure 28: Signals of different breathing states detected by the chemoelectric humidity sensors. For the inhalation process, the current change rate of the sensor is negative due to the lower relative humidity of the ambient air. In contrast, for the exhalation process, the current change rate of the humidity sensor is positive due to the higher relative humidity of the exhaled air. (a) Fast breathing. (b) Normal breathing (Blue: Inhale, Red: Exhale).

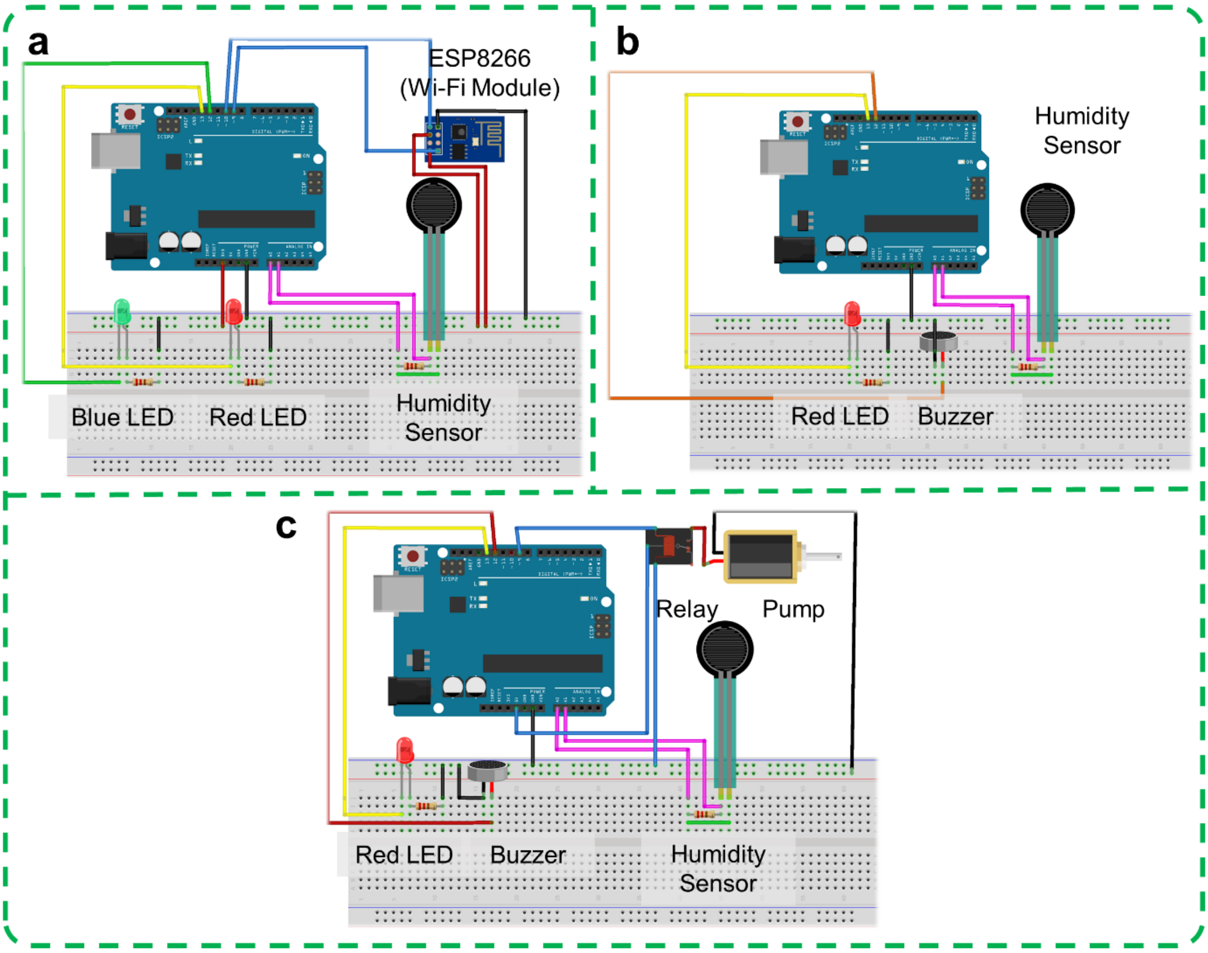

Supplementary Figure 29: Structure of respiratory monitoring-diagnosing-treatment systems. (a) A respiratory monitoring and telemedicine system. (b) A sleep apnea syndrome (SAS) diagnosis and treatment system which can alert people with SAS by buzzer and light-emitting diode (LED). (c) A SAS diagnosis and treatment system that controls ventilator in time by monitoring the breathing status.

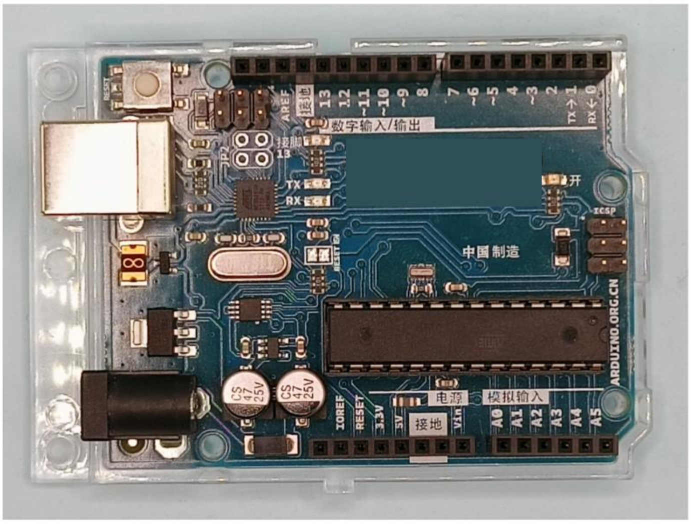

Supplementary Figure 30: Photo of the readout electronics.

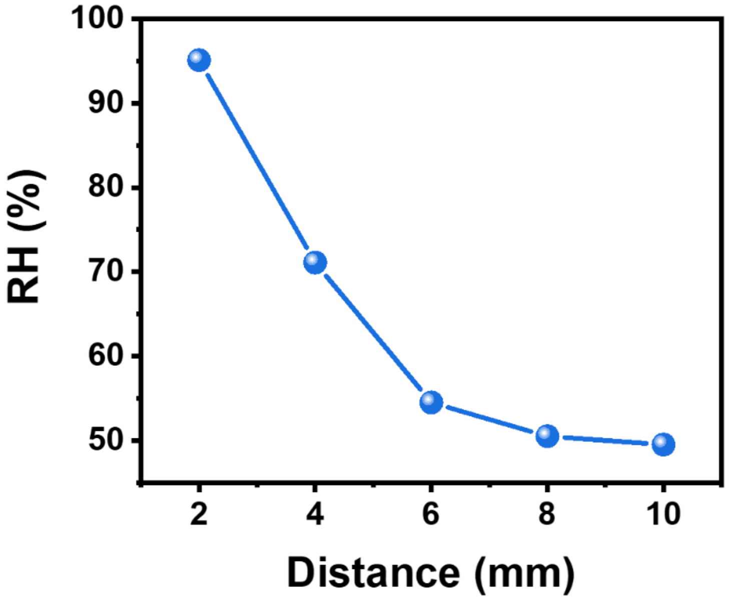

Supplementary Figure 31: Dependence of humidity on distance from the index finger surface. Temperature: 25 °C; relative humidity (RH): 48%.

Supplementary Table 1: Comparison of the response time and recovery time of the our chemoelctric sensors and other reported flexible humidity sensors.

Active Materials	Response Time/s	Recovery Time/s	Mechanism	Ref.
Graphene/Silk	3	6	Resistive	[1]
Graphene Oxide	1.4	10	Resistive	[2]
Graphene Fiber	180	125	Resistive	[3]
Paper	600	1500	Resistive	[4]
Cellulose	6	10.8	Resistive	[5]
MXene	1	201	Resistive	[6]
MXene/Ag	5	80	Resistive	[7]
MXene	1000	2000	Resistive	[8]
Graphene	40	20	Resistive	[9]
PEDOT:PSS	1	4	Resistive	[10]
Graphene Oxide	170	190	Capacitive	[11]
Al/PI	1.5	50.6	Capacitive	[12]
Perovskite	5	5	Capacitive	[13]
Cleancool/Cu fiber	3.5	4	Capacitive	[14]
Graphene Fabrics	120	100	Capacitive	[15]
Graphene Oxide	4.5	5.5	Self-powered	[16]
TiO_2	45	200	Self-powered	[17]
TiO_2	4.5	2.8	Self-powered	[18]
SnS_2/rGO	6	15	Self-powered	[19]
MXene/Cellulose	20	70	Self-powered	[20]
GO/SF	1.05	0.8	Chemoelectric	This Work

GO: graphene oxide, PEDOT:PSS: poly (3,4-ethylenedioxythiophene):poly (styrenesulfonate), PI: polyimide, rGO: reduced graphene oxide, SF: silk fibroin.

References

1. Wang, Q., *et al.* Self-Healable Multifunctional Electronic Tattoos Based on Silk and Graphene. *Advanced Functional Materials* **29**, 1808695 (2019).
2. Wang, X., Xiong, Z., Liu, Z. & Zhang, T. Exfoliation at the Liquid/Air Interface to Assemble Reduced Graphene Oxide Ultrathin Films for a Flexible Noncontact Sensing Device. *Advanced Materials* **27**, 1370-1375 (2015).
3. Choi, S.-J., *et al.* Nitrogen-Doped Single Graphene Fiber with Platinum Water Dissociation Catalyst for Wearable Humidity Sensor. *Small* **14**, 1703934 (2018).
4. Güder, F., *et al.* Paper-Based Electrical Respiration Sensor. *Angewandte Chemie International Edition* **55**, 5727-5732 (2016).
5. Wang, Y., Zhang, L., Zhou, J. & Lu, A. Flexible and Transparent Cellulose-Based Ionic Film as a Humidity Sensor. *ACS Applied Materials & Interfaces* **12**, 7631-7638 (2020).
6. Yang, Z., *et al.* Improvement of Gas and Humidity Sensing Properties of Organ-like MXene by Alkaline Treatment. *ACS Sensors* **4**, 1261-1269 (2019).
7. Liu, L.-X., *et al.* Flexible and Multifunctional Silk Textiles with Biomimetic Leaf-Like MXene/Silver Nanowire Nanostructures for Electromagnetic Interference Shielding, Humidity Monitoring, and Self-Derived Hydrophobicity. *Advanced Functional Materials* **29**, 1905197 (2019).
8. Pazniak, H., *et al.* 2D Molybdenum Carbide MXenes for Enhanced Selective Detection of Humidity in Air. *Advanced Materials* **33**, 2104878 (2021).
9. Lu, L., Jiang, C., Hu, G., Liu, J. & Yang, B. Flexible Noncontact Sensing for Human–Machine Interaction. *Advanced Materials* **33**, 2100218 (2021).
10. Wang, W., *et al.* Inflight fiber printing toward array and 3D optoelectronic and sensing architectures. *Science Advances* **6**, eaba0931 (2020).
11. Ho, D.H., *et al.* Stretchable and Multimodal All Graphene Electronic Skin. *Advanced Materials* **28**, 2601-2608 (2016).
12. Hua, Q., *et al.* Skin-inspired highly stretchable and conformable matrix networks for multifunctional sensing. *Nature Communications* **9**, 244 (2018).
13. Cho, M.-Y., *et al.* Perovskite-Induced Ultrasensitive and Highly Stable Humidity Sensor Systems Prepared by Aerosol Deposition at Room Temperature. *Advanced Functional Materials* **30**, 1907449 (2020).
14. Ma, L., *et al.* Full-Textile Wireless Flexible Humidity Sensor for Human Physiological Monitoring. *Advanced Functional Materials* **29**, 1904549 (2019).
15. Zhao, X., Long, Y., Yang, T., Li, J. & Zhu, H. Simultaneous High Sensitivity Sensing of Temperature and Humidity with Graphene Woven Fabrics. *ACS Applied Materials & Interfaces* **9**, 30171-30176 (2017).
16. Hu, K., *et al.* Self-Powered Electronic Skin with Biotactile Selectivity. *Advanced Materials* **28**, 3549-3556 (2016).
17. Shen, D., *et al.* Self-Powered Wearable Electronics Based on Moisture Enabled Electricity Generation. *Advanced Materials* **30**, 1705925 (2018).
18. Shen, D., *et al.* Self-Powered, Rapid-Response, and Highly Flexible Humidity Sensors Based on Moisture-Dependent Voltage Generation. *ACS Applied Materials & Interfaces* **11**, 14249-14255 (2019).
19. Zhang, D., Xu, Z., Yang, Z. & Song, X. High-performance flexible self-powered tin disulfide nanoflowers/reduced graphene oxide nanohybrid-based humidity sensor driven by triboelectric nanogenerator. *Nano Energy* **67**, 104251 (2020).
20. Li, P., Su, N., Wang, Z. & Qiu, J. A Ti3C2Tx MXene-Based Energy-Harvesting Soft Actuator with Self-Powered Humidity Sensing and Real-Time Motion Tracking Capability. *ACS Nano* **15**, 16811-16818 (2021).
