# Supplementary material for: Humidity-sensitive chemoelectric flexible sensors based on metal-air redox reaction for health management
Source: Nat Commun. 2022 Sep 15;13:5416. doi: 10.1038/s41467-022-33133-y (PMC9477177; doi:10.1038/s41467-022-33133-y)
Supplement: Supplementary file 2 — Description of Additional Supplementary Files [file 41467_2022_33133_MOESM2_ESM.doc]

**Description of Additional Supplementary Files**

**Supplementary Movie 1: Telemedicine.** This video shows the breathing signals are collected through chemoelectric humidity sensor. At the same time, the signals are transmitted to computers and mobile phones through Wi-Fi or 5G, demonstrating the possibility of the sensors for telemedicine.

**Supplementary Movie 2: Respiratory Frequency Detection.** This video shows the breathing signals are collected through chemoelectric sensor. At the same time, the signals are used to control LED through the Arduino. The frequency of the LED light on and off is consistent with the respiratory rate, demonstrating the possibility of the sensor being used for respiratory rate detection.

**Supplementary Movie 3: Sleep Apnea Syndrome Diagnosis and Treatment Through Alarm.** This video shows the breathing signals are collected through chemoelectric sensor and the signals are used to control the buzzer and LED through Arduino. When the subject normal breaths, the LED shows a green light. Once the subject suffers from sleep apnea syndrome, the LED shows a red light and the buzzer alarm, demonstrating the possibility of the sensor being used for sleep apnea syndrome diagnosis and treatment through alarm.

**Supplementary Movie 4: Sleep Apnea Syndrome Diagnosis and Treatment Through Controlling Ventilator.** This video shows the breathing signals are collected through chemoelectric sensor and the signals are used to control the ventilator through Arduino. When the subject normal breaths, the ventilator is powered off. Once the subject suffers from sleep apnea syndrome, the ventilator is powered on, demonstrating the possibility of the sensor being used for sleep apnea syndrome diagnosis and treatment through controlling ventilator.

**Supplementary Movie 5: Non-contact Human-machine Interaction.** This video shows the humidity signals around the chemoelectric sensor are collected and the signals are used to control the elevator through Arduino. When the finger approaches the sensor, the humidity around the sensor increases and the elevator model is powered on. When the sensor is away from the sensor, the elevator model is powered off.
