# Supplementary material for: Humidity-sensitive chemoelectric flexible sensors based on metal-air redox reaction for health management
Source: Nat Commun. 2022 Sep 15;13:5416. doi: 10.1038/s41467-022-33133-y (PMC9477177; doi:10.1038/s41467-022-33133-y)
Supplement: Supplementary file 8 — Reporting Summary [file 41467_2022_33133_MOESM8_ESM.pdf]

nature portfolio

Corresponding author(s): Yingying Zhang

Last updated by author(s): Aug 11, 2022

Reporting Summary

Nature Portfolio wishes to improve the reproducibility of the work that we publish. This form provides structure for consistency and transparency in reporting. For further information on Nature Portfolio policies, see our Editorial Policies and the Editorial Policy Checklist.

Statistics

For all statistical analyses, confirm that the following items are present in the figure legend, table legend, main text, or Methods section.

n/a	Confirmed	
☐	☒	The exact sample size (n) for each experimental group/condition, given as a discrete number and unit of measurement
☐	☒	A statement on whether measurements were taken from distinct samples or whether the same sample was measured repeatedly
☒	☐	The statistical test(s) used AND whether they are one- or two-sided<br>*Only common tests should be described solely by name; describe more complex techniques in the Methods section.*
☒	☐	A description of all covariates tested
☒	☐	A description of any assumptions or corrections, such as tests of normality and adjustment for multiple comparisons
☐	☒	A full description of the statistical parameters including central tendency (e.g. means) or other basic estimates (e.g. regression coefficient) AND variation (e.g. standard deviation) or associated estimates of uncertainty (e.g. confidence intervals)
☒	☐	For null hypothesis testing, the test statistic (e.g. F, t, r) with confidence intervals, effect sizes, degrees of freedom and P value noted<br>*Give P values as exact values whenever suitable.*
☒	☐	For Bayesian analysis, information on the choice of priors and Markov chain Monte Carlo settings
☒	☐	For hierarchical and complex designs, identification of the appropriate level for tests and full reporting of outcomes
☒	☐	Estimates of effect sizes (e.g. Cohen's d, Pearson's r), indicating how they were calculated

Our web collection on statistics for biologists contains articles on many of the points above.

Software and code

Policy information about availability of computer code

Data collection	CHI 760E, Arduino IDE (version 1.6.5). The custom codes developed in the study are available at https://github.com/LSTHU/Respiratory_sensing.
Data analysis	Origin 2018 edu version

For manuscripts utilizing custom algorithms or software that are central to the research but not yet described in published literature, software must be made available to editors and reviewers. We strongly encourage code deposition in a community repository (e.g. GitHub). See the Nature Portfolio guidelines for submitting code & software for further information.

Data

Policy information about availability of data

All manuscripts must include a data availability statement. This statement should provide the following information, where applicable:

- Accession codes, unique identifiers, or web links for publicly available datasets
- A description of any restrictions on data availability
- For clinical datasets or third party data, please ensure that the statement adheres to our policy

The data generated in this study have been deposited in the Science Data Banke database under accession code http://cstr.cn/31253.11.sciencedb.01874.

Human research participants

Policy information about studies involving human research participants and Sex and Gender in Research.

Reporting on sex and gender	Two healthy subjects including 1 male and 1 female were involved in the respiratory monitoring test.
Population characteristics	Two healthy subjects were involved in the respiratory monitoring test, whose ages ranged from 22-30.
Recruitment	Subjects were invited by open recruitment in Tsinghua University, to participate this research. Subjects were required to be healthy and had no allergies. The physical state of the subjects is different, thus, the signal of respiratory may be influenced by different individuals.
Ethics oversight	The data was obtained with the informed consent of all participants. The Institutional Review Board of Tsinghua University approved this study (NO. 20220118).

Note that full information on the approval of the study protocol must also be provided in the manuscript.

Field-specific reporting

Please select the one below that is the best fit for your research. If you are not sure, read the appropriate sections before making your selection.

[x] Life sciences [ ] Behavioural & social sciences [ ] Ecological, evolutionary & environmental sciences

For a reference copy of the document with all sections, see nature.com/documents/nr-reporting-summary-flat.pdf

Life sciences study design

All studies must disclose on these points even when the disclosure is negative.

Sample size	Two biologically independent samples were used for each test. The related experiments are only used to demonstrate the possibility of the sensor for respiration monitoring, therefore two biologically independent samples are sufficient.
Data exclusions	No data exclusion involved.
Replication	Data were measured at least three times to make sure that data are reproducible. All attempts for replication are successful.
Randomization	No randomization was used. We analyzed all the results of the test. The related experiments are only used to demonstrate the possibility of the sensor for respiration monitoring, therefore randomization is not applicable to this study.
Blinding	No blinding is necessary since we analyzed all the respiratory data collected with the help of the subjects.

Reporting for specific materials, systems and methods

We require information from authors about some types of materials, experimental systems and methods used in many studies. Here, indicate whether each material, system or method listed is relevant to your study. If you are not sure if a list item applies to your research, read the appropriate section before selecting a response.

Materials & experimental systems

n/a	Involved in the study	
[x]	[ ]	Antibodies
[x]	[ ]	Eukaryotic cell lines
[x]	[ ]	Palaeontology and archaeology
[x]	[ ]	Animals and other organisms
[x]	[ ]	Clinical data
[x]	[ ]	Dual use research of concern

Methods

n/a	Involved in the study	
[x]	[ ]	ChIP-seq
[x]	[ ]	Flow cytometry
[x]	[ ]	MRI-based neuroimaging
